# Severe COVID-19 Is Associated With an Altered Upper Respiratory Tract Microbiome

**DOI:** 10.3389/fcimb.2021.781968

**Published:** 2022-01-24

**Authors:** Meghan H. Shilts, Christian Rosas-Salazar, Britton A. Strickland, Kyle S. Kimura, Mohammad Asad, Esha Sehanobish, Michael H. Freeman, Bronson C. Wessinger, Veerain Gupta, Hunter M. Brown, Helen H. Boone, Viraj Patel, Mali Barbi, Danielle Bottalico, Meaghan O’Neill, Nadeem Akbar, Seesandra V. Rajagopala, Simon Mallal, Elizabeth Phillips, Justin H. Turner, Elina Jerschow, Suman R. Das

**Affiliations:** ^1^ Department of Medicine, Vanderbilt University Medical Center, Nashville, TN, United States; ^2^ Department of Pediatrics, Vanderbilt University Medical Center, Nashville, TN, United States; ^3^ Department of Pathology, Microbiology and Immunology, Vanderbilt University Medical Center, Nashville, TN, United States; ^4^ Department of Otolaryngology-Head and Neck Surgery, Vanderbilt University Medical Center, Nashville, TN, United States; ^5^ Department of Medicine, Montefiore Medical Center/Albert Einstein College of Medicine, Bronx, NY, United States

**Keywords:** SARS-CoV-2, COVID-19, upper respiratory tract, microbiome, mild, moderate, severe COVID-19 outcomes

## Abstract

**Background:**

The upper respiratory tract (URT) is the portal of entry of severe acute respiratory syndrome coronavirus 2 (SARS-CoV-2), and SARS-CoV-2 likely interacts with the URT microbiome. However, understanding of the associations between the URT microbiome and the severity of coronavirus disease 2019 (COVID-19) is still limited.

**Objective:**

Our primary objective was to identify URT microbiome signature/s that consistently changed over a spectrum of COVID-19 severity.

**Methods:**

Using data from 103 adult participants from two cities in the United States, we compared the bacterial load and the URT microbiome between five groups: 20 asymptomatic SARS-CoV-2-negative participants, 27 participants with mild COVID-19, 28 participants with moderate COVID-19, 15 hospitalized patients with severe COVID-19, and 13 hospitalized patients in the ICU with very severe COVID-19.

**Results:**

URT bacterial load, bacterial richness, and within-group microbiome composition dissimilarity consistently increased as COVID-19 severity increased, while the relative abundance of an amplicon sequence variant (ASV), *Corynebacterium*_unclassified.ASV0002, consistently decreased as COVID-19 severity increased.

**Conclusions:**

We observed that the URT microbiome composition significantly changed as COVID-19 severity increased. The URT microbiome could potentially predict which patients may be more likely to progress to severe disease or be modified to decrease severity. However, further research in additional longitudinal cohorts is needed to better understand how the microbiome affects COVID-19 severity.

## Introduction

The upper respiratory tract (URT) microbiome is an important contributor to respiratory health (Man et al., 2017). The URT microbiome can impact both short- and long-term clinical outcomes of common respiratory viruses (including acute disease severity) (de Steenhuijsen Piters et al., 2016; Rosas-Salazar et al., 2018; Sonawane et al., 2019), as well as viral load (Ederveen et al., 2018), acute immune response (Ederveen et al., 2018; Shilts MH et al., 2020),, and host gene expression patterns (de Steenhuijsen Piters et al., 2016; Sonawane et al., 2019) associated with these viruses. During the last major influenza pandemic in 1918–1919, more people died due to secondary bacterial infections than due to the virus (Morens et al., 2008). However, understanding of the association of the URT microbiome with clinical outcomes related to severe acute respiratory syndrome coronavirus-2 (SARS-CoV-2)—the respiratory virus responsible for the ongoing coronavirus disease 2019 (COVID-19) pandemic—is limited, despite the URT being a major portal of entry for this virus (Gallo et al., 2020). While previous research has focused mostly on comparing the respiratory microbiome during COVID-19 to uninfected controls (De Maio et al., 2020; Shen et al., 2020; Zhang et al., 2020; Haiminen et al., 2021; Maes et al., 2021; Nardelli et al., 2021; Rosas-Salazar et al., 2021; Xu et al., 2021), studies examining the association between the URT microbiome and COVID-19 severity have been limited thus far (Mostafa et al., 2020; Hernández-Terán et al., 2021; Li et al., 2021; Merenstein et al., 2021; Rueca et al., 2021; Ventero et al., 2021). To start filling this gap in knowledge, we compared the URT microbiome among uninfected adults and adults with mild, moderate, severe [but without the necessity for intensive care unit (ICU) admission], and very severe (admitted to the ICU) COVID-19. We hypothesized that URT microbiome signatures would be associated with an increase or decrease in COVID-19 severity.

## Methods

### Study Cohorts and Sample Collection

URT swabs were collected during spring 2020 from 114 adult participants enrolled at two separate sites: 1) 86 participants were enrolled in Nashville, Tennessee: 65 with mild-to-moderate COVID-19 and 21 asymptomatic SARS-CoV-2 uninfected controls, and 2) 28 participants were enrolled in Bronx, New York, who were hospitalized due to severe COVID-19. In all participants, SARS-CoV-2 infection was tested by RT-qPCR.

The 65 participants who had mild-to-moderate COVID-19 were enrolled as part of a randomized clinical trial conducted at Vanderbilt University Medical Center (VUMC) to investigate the effect of nasal irrigations on disease course, as previously described (Kimura et al., 2020). These participants were all seen on an ambulatory basis and none were hospitalized. Concurrently, 21 SARS-CoV-2 RT-qPCR negative adults without COVID-19 or other acute respiratory disease symptoms were recruited from the VUMC community (clinicians, students, faculty, and staff). All participants enrolled at VUMC were given swabs and viral preservation media and performed mid-turbinate swabs as directed; only the enrollment swabs were included in this analysis. Informed consent was obtained from all participants, and this study was approved by the VUMC Institutional Review Board (IRB).

Nasopharyngeal swabs were obtained from patients with COVID-19-like symptoms admitted to the Montefiore Medical Center in the Bronx, New York, by hospital staff at admission. Leftover test swab aliquots in viral preservation media from 28 patients who tested positive for SARS-CoV-2 were sent to VUMC for further laboratory processing. All patients or their surrogate decision-makers signed an informed consent at enrollment, which was approved by the IRB at Albert Einstein College of Medicine.

### Characterization of the Upper Respiratory Microbiome

Further details are available in the **Online Supplement**. To characterize the URT microbiome, all samples were processed at VUMC with the same method. DNA was extracted with the PowerSoil HTP Kit (Qiagen), the target hypervariable region V4 of the 16S ribosomal RNA (rRNA) gene was amplified with previously published primers to construct libraries (Kozich et al., 2013), and libraries were pooled and then sequenced on an Illumina MiSeq with 2 × 250 bp reads (Shilts M et al., 2020). A ZymoBIOMICS mock community controls (Zymo) and 19 negative controls were processed concurrently with the samples. Sequenced reads were run in R version 4.0.3 through *dada2* (Callahan et al., 2016) version 1.18.0 to remove low-quality reads, construct amplicon sequence variants (ASVs), and assign taxonomy against the SILVA reference database (Pruesse et al., 2007). Potential contaminants were removed with the “prevalence” method in *decontam* (Davis et al., 2018) version 1.10.0. Samples with <1,000 reads were removed (*N* = 7). For ASVs of interest, if the species was not determined with the *dada2* workflow, we used the standard nucleotide basic local alignment search tool (BLAST) to search its sequence against the NCBI 16S rRNA sequences (Bacteria and Archaea) database, excluding uncultured/environmental sample sequences, available at https://blast.ncbi.nlm.nih.gov/Blast.cgi. Sequences were deposited to the Sequence Read Archive at NCBI under BioProjects PRJNA726992 and PRJNA726994.

Bacterial load was assessed using universal 16S rRNA primers as previously described (Barman et al., 2008). Prior to analysis, bacterial copy number was log transformed.

### Creation of Preselected COVID-19 Severity Groups

As we had data from two separate sites (Tennessee and New York), and URT sampling methods differed between these two sites (mid-turbinate self-swab versus healthcare provider-performed nasopharyngeal swab, respectively), we looked for microbiome trends that were robust to differences in sites and sampling methods. Therefore, we split the samples from the two sites into five prespecified severity groups (uninfected controls and mild-, moderate-, severe-, and very severe-SARS-CoV-2-infected) to examine if changes in the URT microbiome were consistent over a spectrum of disease severities. As described below, severity groups were chosen to be both clinically relevant and to have an approximately even sample size from each site.

The participants from Tennessee who did not have COVID-19 or other respiratory illness symptoms and were SARS-CoV-2 negative by RT-qPCR were designated the “uninfected” control group (*N* = 20).

All SARS-CoV-2-infected participants from Tennessee, who had mild-to-moderate illness and were not hospitalized, were given a symptom score questionnaire. At enrollment, participants were asked to rate their symptoms over the last 24 h on an ordinal scale, with 0 indicating the symptom was not present and 7 indicating the symptom was the most severe. Symptoms included were cough, eye redness, nasal congestion, headache, sore throat, sputum production, fatigue, coughing blood, shortness of breath, nausea/vomiting, diarrhea, muscle or joint pain, chills, loss of smell or taste, loss of the ability to think clearly, inability to sleep well, and inability to breathe easily. Summed symptom scores ranged from 0 to 78, with a median (interquartile range) of 30 (15.5–44.5); two participants did not fill out the symptom score questionnaire and so were excluded (**Figure E1**). Those with a summed symptom score under the median were labeled the “mild” COVID-19 group (*N* = 27) and those with a summed symptom score at or over the median were labeled the “moderate” COVID-19 group (*N* = 28).

All SARS-CoV-2-infected participants from New York were hospitalized. Within this group, patients were divided into the “severe” group, who were not admitted to the ICU (*N* = 15), and the “very severe” group, who were admitted to the ICU (*N* = 13).

A summary of participant samples included/excluded can be found in **Figure E1**. Descriptive statistics were used to characterize the five disease severity groups for the participants who were included in the analysis. A Kruskal–Wallis, Wilcoxon rank-sum, or Pearson chi-squared test, as appropriate, was used to test for differences in variables between the groups.

### Statistical Analysis

Microbiome data processing, as described below, was performed in R version 3.5.1 using the wrapper MGSAT (Tovchigrechko, 2020). ASV abundances within each sample were normalized using simple proportions. ASVs with average absolute counts <10 and those with an average relative abundance <0.0005 were aggregated into category “other” which was used when calculating relative abundances but otherwise disregarded. Ninety-five ASVs remained after this filtering. The R package *vegan* (Oksanen et al., 2014) version 2.5-2 was used to calculate richness and alpha- and beta-diversity at the ASV level. Hill numbers N0, N1, and N2 were used to assess, respectively, richness, the exponential Shannon index, and the inverted Simpson alpha-diversity index (Hill, 1973). Pairwise differences in microbial community composition between all samples were assessed with the Bray–Curtis dissimilarity index, computed on simple proportions, and the PERMANOVA test as implemented in *adonis2* (Anderson, 2001) was used to test for significant differences between overall microbial composition and severity groups; age and sex were added to the model. A Tukey HSD *post-hoc* test was run to examine the significance of pairwise group comparisons. The *betadisper* function in *vegan* was used to test for differences in variance between the groups. Variance in Bray–Curtis dissimilarities between each severity group was examined further with the Kruskal–Wallis test and ordinal logistic regression, as described below.

In addition to bacterial load, we preselected the following variables extracted from the bacterial 16S rRNA microbiome data to apply the Kruskal–Wallis test for significant differences between the COVID-19 severity groups: richness, Shannon alpha-diversity, Simpson alpha-diversity, pairwise Bray–Curtis dissimilarities within each severity group, and the 95 ASVs that remained after filtering. The Benjamini–Hochberg correction was applied to adjust for multiple comparisons. Eta-squared (effect size) and its 95% confidence intervals (CI) were found over 1,000 bootstrap replications with the *kruskal_effsize* function in the R package *rstatix* version 0.6.0 (Tomczak and Tomczak, 2014; Kassambara, 2020).

To further explore how the microbiome changed as COVID-19 severity increased, we next performed ordinal logistic regression with the *lrm* function in the R package *rms* (version 6.2-0) (Harrell, 2021), setting the COVID-19 severity groups as the dependent variable and the microbiome components as the independent variables. Interquartile odds ratios (OR) and their 95% CIs were found with *rms*::*summary.rms* and *P*-values for each independent variable were calculated with the *rms*::*anova* function.

As we were interested only in microbiome parameters that consistently increased/decreased as disease severity increased, to minimize overfitting the models, and because some of the microbiome variables may have high collinearity due to how they are calculated (e.g., the richness, Shannon, and Simpson alpha-diversity indices assess a similar phenomenon; the ASV relative abundances could be correlated due to normalization), we applied a strict selection criteria as to which microbiome variables would be added to the ordinal logistic regression models. In addition to bacterial load, microbiome variables were only added to the model if 1) the adjusted Kruskal–Wallis test result *P <*0.1 and 2) its median either consistently increased or decreased as COVID-19 severity increased.

Age, sex, race, presence of comorbidities, and current smoking were *a priori* selected to be added to the model as independent variables due to their associations with COVID-19 severity. The race/ethnicity categories were simplified to Black, Hispanic, White, or Other. Comorbidities were reduced to presence of any comorbidity [comorbidities included obesity (defined as a body mass index > 30), diabetes, hypertension, heart disease, or lung disease]. Due to the small number of participants on antibiotics (*N* = 2) or inhaled steroids (*N* = 1), we did not add these variables to any models.

Two different main ordinal logistic regression models were built, containing as the independent variables the following: model 1—age, sex, presence of any comorbidities, current smoking, race, bacterial load, bacterial richness, and relative abundance of *Corynebacterium*_unclassified.ASV0002 and model 2—within-group pairwise Bray–Curtis dissimilarities, which had to be tested separately from all the other variables due to fundamental differences in data structure. The following data were missing for some participants: race/ethnicity (*N* = 9), comorbidities (*N* = 3), current smoking (*N* = 1), and bacterial load (*N* = 6). We first ran ordinal logistic regression for model 1 (designated model 1A) with only the complete cases (*N* = 87). Next, we imputed the missing data over 20 imputations with *Hmisc (* Harrell and , with contributions from Charles Dupont and many others, 2020) (version 4.4.2) function *aregImpute* and ran ordinal logistic regression (designated model 1B) using *Hmisc*::*fit.mult.impute* with the imputed data so that all cases were included (*N* = 103). Further details are available in the **Online Supplement**.

Figures were generated with the R package *ggplot2* (Wickham, 2009) version 3.3.3.

## Results

### Sample Inclusion/Exclusion and Participant Demographics and Clinical Characteristics

A flowchart showing participant samples that were retained for analysis can be found in **Figure E1**. Demographic and clinical characteristics of the 103 study participants whose samples were included for analysis can be found in **Table 1**. The hospitalized participants (severe or very severe COVID-19) tended to be older, were more likely to be Black or Hispanic, and had a comorbidity rate higher than the non-hospitalized participants. Among hospitalized patients, those who were admitted to the ICU had a higher mortality rate, were more likely to be placed on a ventilator, and had longer hospital stays than those who were not admitted to the ICU. As the incidence of similar symptoms was captured between both cohorts, tables reporting symptom status by COVID-19 severity (**Table E1**) and age quartile (**Table E2**) are available in the **Online Supplement**.

**Table 1:** Baseline and clinical characteristics of SARS-CoV-2 uninfected controls and infected study participants included in the analysis.

Characteristic	All (*N* = 103)	Uninfected controls (*N* = 20)	COVID mild (*N* = 27)	COVID moderate (*N* = 28)	COVID severe (*N* = 15)	COVID very severe (*N* = 13)	*P*-value^a^
Baseline characteristics							
Age (years)	41 (29–58)	31 (29–39)	49 (31–63)	33 (27–40)	58 (51–73)	55 (52–60)	<0.001
Male sex	53 (52%)	12 (60%)	15 (56%)	13 (46%)	5 (33%)	8 (62%)	0.47
Race/ethnicity							<0.001
American Indian	1 (1%)	0	0	0	0	1 (8%)	
Asian	5 (5%)	3 (15%)	0	0	1 (7%)	1 (8%)	
Black	17 (17%)	0	5 (19%)	1 (4%)	5 (33%)	6 (46%)	
Hispanic	12 (12%)	0	1 (4%)	0	7 (5%)	4 (31%)	
White	59 (57%)	17 (85%)	15 (56%)	25 (89%)	1 (7%)	1 (8%)	
Unknown	9 (9%)	0	6 (22%)	2 (7%)	0	1 (8%)	
Recent use of antibiotics	2 (2%)	0	0	0	1 (7%)	1 (8%)	0.17
Current use of intranasal medications	1 (1%)	0	0	0	0	1 (8%)	0.14
Current smoker	5 (5%)	1 (5%)	1 (4%)	0	3 (20%)	0	0.05
Obese (BMI > 30)	28 (27%)	4 (20%)	5 (19%)	8 (29%)	5 (33%)	6 (46%)	0.78
Diabetes	12 (12%)	0	1 (4%)	3 (11%)	5 (33%)	3 (23%)	0.01
Hypertension	28 (27%)	2 (10%)	7 (26%)	3 (11%)	9 (60%)	7 (54%)	<0.001
Lung disease	12 (12%)	0	5 (19%)	3 (11%)	1 (7%)	3 (23%)	0.22
Heart disease	19 (18%)	1 (5%)	2 (7%)	0	9 (60%)	7 (54%)	<0.001
Clinical characteristics							
Symptom score^b^		NA	15 (12–24)	44.5 (32.8–55)	NA	NA	<0.001
Length of hospital stay (days)^c^	12 (4.5–31.5)	0	0	0	4.5 (3–7.75)	32 (17–44)	<0.001
Patient on ventilator	11 (11%)	0	0	0	0	11 (85%)	<0.001
Patient deceased	6 (6%)	0	0	0	1 (7%)	5 (39%)	<0.001

The data are presented as median (interquartile range) for continuous variables or number (%) for categorical variables. Except for race/ethnicity, the estimates were calculated for participants with complete data.

SARS-CoV-2, severe acute respiratory syndrome coronavirus-2; BMI, body mass index.

a P-value for the comparison between groups using Kruskal–Wallis or Pearson’s chi-squared test, as appropriate.

b P-value was calculated with a Wilcoxon rank-sum test with continuity correction only between the mild and moderate severity groups. Severity scores were obtained by asking the patients to rank their symptoms, with higher values indicating more severe disease. Uninfected control participants and hospitalized patients were not asked to fill out this symptom score questionnaire.

c P-value was calculated with a Wilcoxon rank-sum test with continuity correction only between the two groups (severe and very severe COVID-19) in which patients were admitted to the hospital. NA, not applicable.

### Overall Microbiome Community Composition Summary

**Figure 1:**
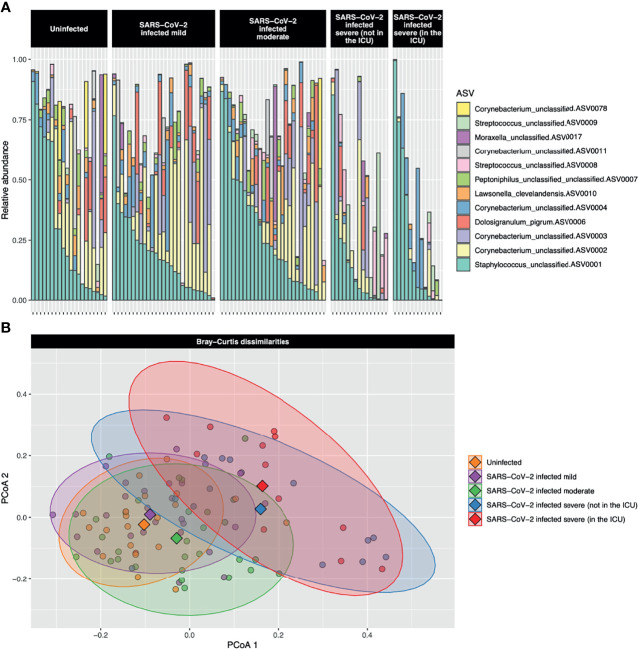
**(A)** Stacked bar charts of the relative abundance of the 12 most abundant amplicon sequence variants (ASVs) are shown for each study participant. The most abundant ASV, *Staphylococcus*_unclassified.ASV0001, was abundant both in uninfected controls and participants with the full range of COVID-19 severities. The second most abundant ASV, *Corynebacterium*_unclassified.ASV0002, was highly abundant in uninfected control participants and those with mild-to-moderate COVID-19, but was of low abundance in those with severe or very severe COVID-19. **(B)** A principal coordinate analysis (PCoA) plot of the Bray–Curtis dissimilarities over the first two axes is shown. Dots represent individual data points and diamonds show the centroids. The 90% confidence data ellipses are shown for each of the COVID-19 severity groups. Overall, microbial community composition was significantly dissimilar among the severity groups (*P* < 0.001).

Abundant ASVs were similar among the five severity groups (**Figure 1A**). The most abundant ASVs were *Staphylococcus*_unclassified.ASV0001 (23.6%), *Corynebacterium*_unclassified.ASV0002 (13.6%), *Corynebacterium*_unclassified.ASV0003 (7.0%), *Dolosigranulum_pigrum*.ASV0006 (4.1%), and *Corynebacterium*_unclassified.ASV0004 (4.5%). However, when all ASVs were included, bacterial community composition (PERMANOVA *P* < 0.001, *R^2^* = 0.08) and dispersion (*betadisper* test *P* = 0.005) differed among the severity groups (**Figure 1B**). Along principal coordinate analysis (PCoA) axis 1 (explaining 7.6% of the variance), the severity groups consistently moved along the same gradient (**Figures 1B** and **E2**). A Tukey HSD *post-hoc* test was run to examine group centroid pairwise comparisons: uninfected control participants compared with the very severe COVID-19 patients were significant [adjusted *P* = 0.005, difference (95% CI) = 0.08 (0.02–0.14)], while mild compared to very severe (adjusted *P* = 0.054, difference (95% CI) = 0.06 (−0.001 to 0.12)) and uninfected controls compared with severe [adjusted *P* = 0.06 difference (95% CI) = 0.06 (−0.002 to 0.12))] approached significance.

### Summary of Kruskal–Wallis and Ordinal Logistic Regression Testing Results

The full Kruskal–Wallis results testing for significant differences of the preselected microbiome parameters (i.e., alpha-diversity indices, beta-diversity, bacterial load, and all ASVs passing the abundance cutoff) among the COVID-19 severity groups are available in **Table E3**. Only bacterial richness, the within-group Bray–Curtis dissimilarities, and 28 ASVs had an adjusted *P <*0.1. Of these 30 microbiome parameters passing the adjusted *P*-value cutoff, only bacterial richness, the within-group Bray–Curtis dissimilarities, and *Corynebacterium*_unclassified.ASV0002 had medians that consistently increased or decreased as COVID-19 severity increased. These three microbiome parameters, along with bacterial load, were examined further with ordinal logistic regression.

Our results were similar regardless of whether we used only complete cases (model 1A) or the imputed dataset (model 1B); therefore, only the results with the complete cases (model 1A) are presented throughout the rest of the manuscript. The full results are available in the **Online Supplement** and **Tables E4, E5** and **Figures E3**–**5**.

Due to their association with COVID-19 severity, age, sex, presence of comorbidities, and current smoking were added to ordinal logistic regression model 1. While increased age [*P* = 0.65, OR (95% CI) 1.2 (0.55–2.59)], female sex [*P* = 0.32, OR (95% CI) 1.51 (0.67–3.38)], and presence of comorbidities [*P* = 0.26, OR (95% CI) 1.9 (0.77–4.69)] were associated with increased disease severity, and current smoking was associated with a reduced risk [*P* = 0.65, OR (95% CI) 0.61 (0.07–5.07)], only race was significantly associated with COVID-19 severity [*P* = 0.02, respective ORs (95% CIs) for Black : White, Hispanic : White, and Other : White were 5.31 (1.24–22.73), 10.8 (2.29–50.83), and 3.19 (0.48–21.04)]. Ordinal logistic regression results for each of the tested microbiome parameters are described in detail below.

### Increased Bacterial Load Was Associated With COVID-19 Severity

**Figure 2:**
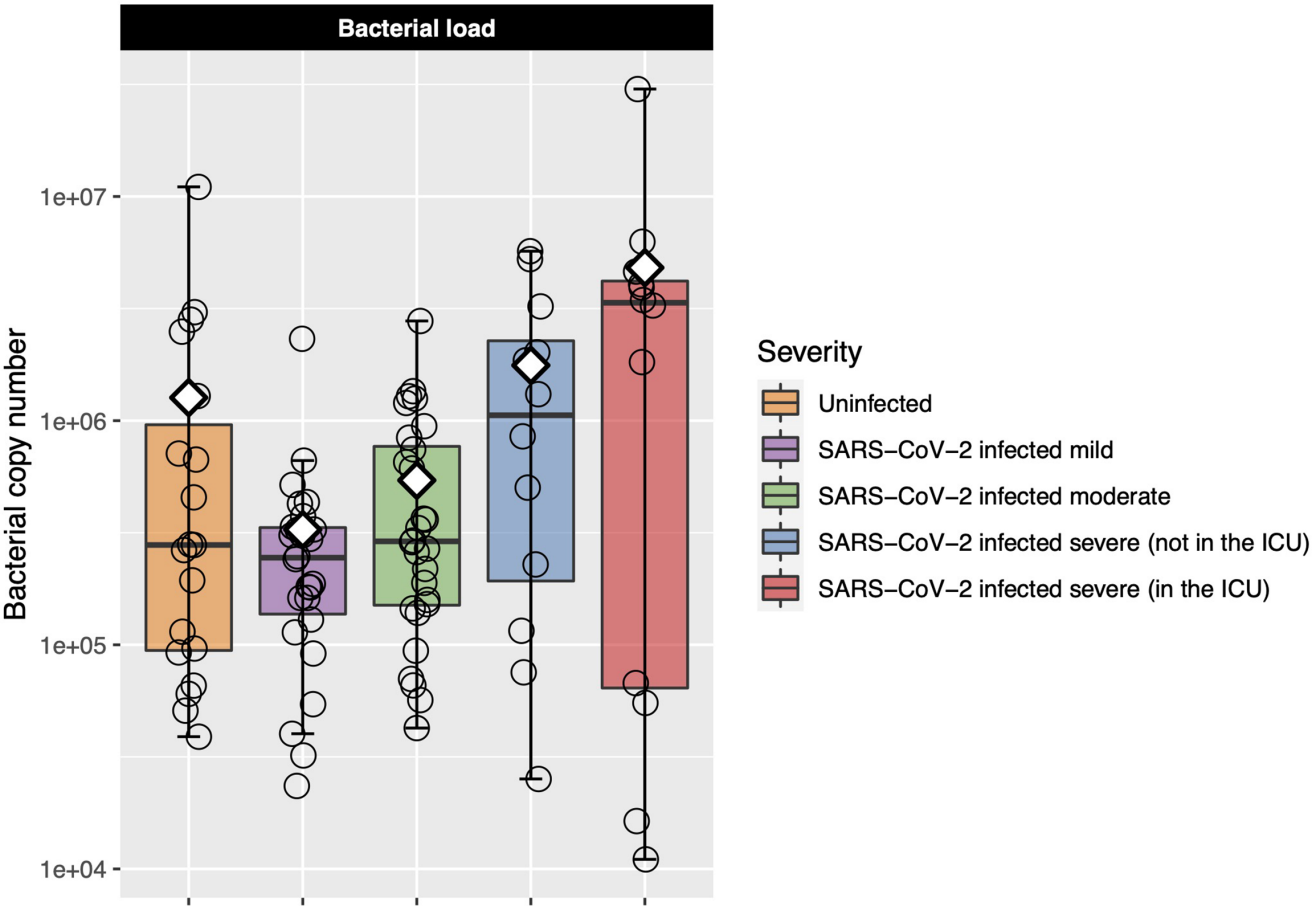
Log-transformed bacterial load is shown for each of the COVID-19 severity groups. Each box represents the median and interquartile range, and the mean is shown by the white diamond. Individual points are shown as open circles. Bacterial load of the uninfected control participants was similar to that of those with mild-to-moderate COVID-19. Among those with COVID-19, there was a trend toward increasing bacterial load as disease severity increased.

Six samples failed the bacterial load assay and so were excluded from analysis. Bacterial load was similar in the uninfected controls and mild and moderate COVID-19 participants (**Figure 2**). Among only those infected with SARS-CoV-2, bacterial load increased as COVID-19 severity increased, although differences between groups were not statistically significant [Kruskal–Wallis adjusted *P* = 0.23, eta-squared (95% CI) = 0.04 (−0.02 to 0.24)] (**Figure 2** and **Table E3**). However, when we performed ordinal logistic regression, bacterial load was significantly associated with disease severity [*P* = 0.04, OR (95% CI) = 2.09 (1.04–4.21)].

### Increased Bacterial Richness and Dissimilarity Within Groups Were Associated With COVID-19 Severity

**Figure 3:**
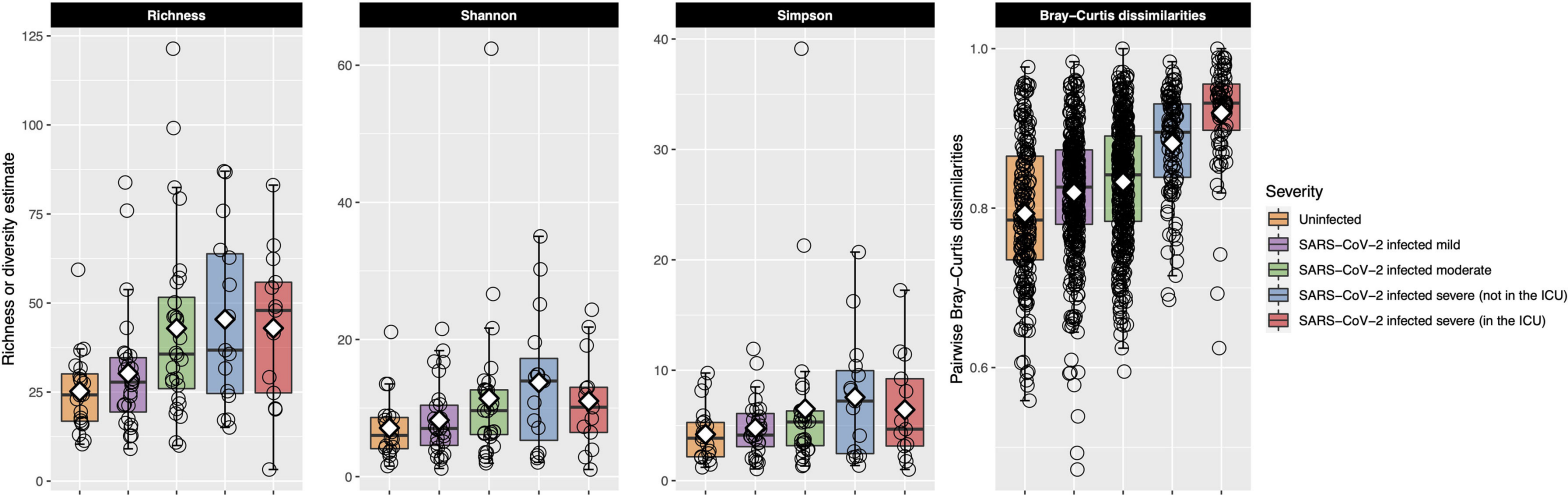
Bacterial richness and alpha- and beta-diversity results are plotted for each of the COVID-19 severity groups. Each box represents the median and interquartile range, and the mean is shown by the white diamond. Individual points are shown as open circles. In the first three facets, bacterial richness and alpha-diversity are shown for each of the patient groupings. Richness and alpha-diversity (Shannon and Simpson indices) were lowest in the uninfected control participants and generally increased as COVID-19 severity increased. Alpha-diversity (Shannon and Simpson indices) decreased in patients in the ICU with very severe COVID-19. The last facet shows within-group pairwise Bray–Curtis dissimilarities plotted on the *y*-axis for each of the COVID-19 groups. Larger values indicate that the URT microbial community between the two samples was more dissimilar, while smaller values indicate the opposite. Uninfected control participants had the most similar URT microbiome to each other, while the URT microbiome within each group became more dissimilar as COVID-19 severity increased. The URT microbiomes among those with very severe COVID-19 were very dissimilar to each other.

Bacterial richness and alpha-diversity generally increased as disease severity increased, although alpha-diversity dropped in patients with very severe COVID-19 (**Figure 3**). This change in richness was near significant with the Kruskal–Wallis test [adjusted *P* = 0.050, eta-squared (95% CI) = 0.09 (0.01–0.27)], while neither the Shannon nor Simpson indices were significant (**Table E3**). Richness was further examined with ordinal logistic regression, and it was near significantly associated with COVID-19 severity [*P* = 0.08, OR (95% CI) = 1.51 (0.96–2.37)].

After calculating pairwise Bray–Curtis dissimilarities between all samples and examining only the within-group pairs, we observed that dissimilarity between samples increased as COVID-19 severity increased (**Figure 3**). Within-group dissimilarities were significantly different with a Kruskal–Wallis test [adjusted *P* < 0.001, eta-squared (95% CI) = 0.16 (0.13–0.21)] and were significantly associated with COVID-19 severity in our ordinal logistic regression model [*P* < 0.001, OR (95% CI) = 2.69 (2.28–3.17)]. When we looked at all Bray–Curtis dissimilarities, within-group dissimilarities in the very severe COVID-19 group were among the highest, similar to the dissimilarities between different COVID-19 severity groups (**Figure E6**).

### A *Corynebacterium* ASV Decreased in Abundance With Increased COVID-19 Severity

**Figure 4:**
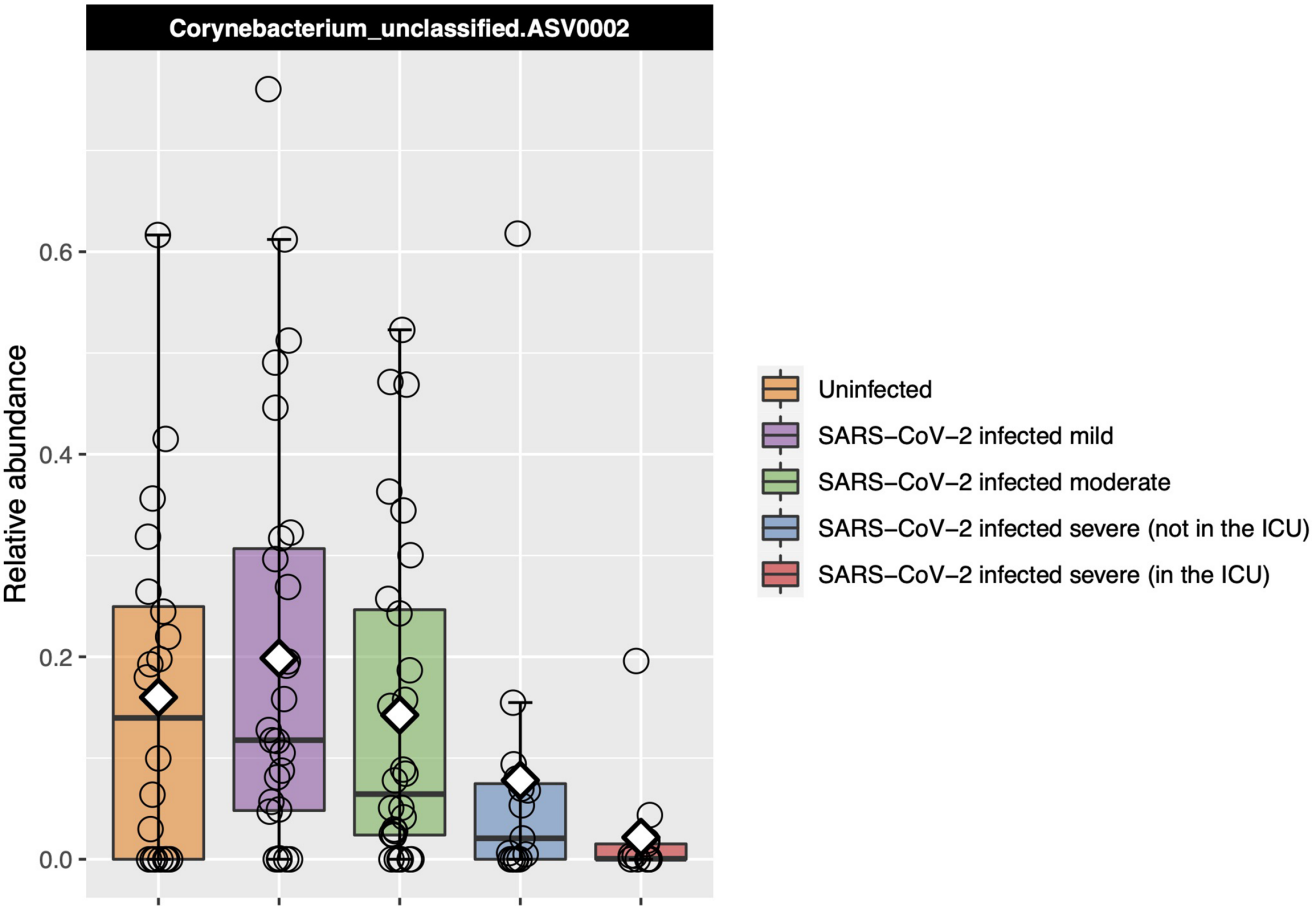
Relative abundance of the one ASV that was identified as significantly differentially abundant by Kruskal–Wallis testing between the COVID-19 groups and that also had a median which consistently changed as COVID-19 severity increased. Each box represents the median and interquartile range, and the mean is shown by the white diamond. Individual points are shown as open circles. *Corynebacterium*_unclassified.ASV0002 abundance decreased as disease severity increased.

*Corynebacterium*_unclassified.ASV0002 relative abundance was found to be significantly different between severity groups with Kruskal–Wallis test [adjusted *P* = 0.04, eta-squared (95% CI) = 0.10 (0.03–0.26)], decreasing as disease severity increased (**Figure 4**). However, in our ordinal logistic regression model, *Corynebacterium*_unclassified.ASV0002 relative abundance was not significantly associated with disease severity [*P* = 0.11 and OR (95% CI) = 0.69 (0.42–1.09)]. The full-length sequence matched with 100% identity to both *Corynebacterium accolens* and *Corynebacterium macginleyi*.

## Discussion

While data on bacterial co-infections in patients with COVID-19 are exponentially increasing during the first year of the pandemic (Lansbury et al., 2020), there are still gaps in our knowledge as to whether the respiratory microbiome plays a significant role in COVID-19 severity and outcomes. The URT microbiome is an important contributor to respiratory health (Man et al., 2017), impacts the severity of other respiratory viruses (de Steenhuijsen Piters et al., 2016; Rosas-Salazar et al., 2018; Sonawane et al., 2019), and can influence acute immune response (Ederveen et al., 2018; Shilts et al., 2020), and the URT is a major portal of entry for this virus (Gallo et al., 2020). To better understand how the URT microbiome could impact COVID-19 severity and outcomes, our study showed that URT bacterial load, richness, and within-group dissimilarity increased, while the relative abundance of a *Corynebacterium* ASV decreased, as COVID-19 severity increased.

Previous studies have examined the association of SARS-CoV-2 infection with the respiratory microbiome (De Maio et al., 2020; Mostafa et al., 2020; Shen et al., 2020; Zhang et al., 2020; Haiminen et al., 2021; Maes et al., 2021; Nardelli et al., 2021; Rosas-Salazar et al., 2021; Rueca et al., 2021; Ventero et al., 2021; Xu et al., 2021), focusing mostly on comparing SARS-CoV-2-infected and uninfected control patients, rather than on associations of the URT microbiome with disease severity among those with SARS-CoV-2. However, there have been studies that have examined the URT microbiome over a spectrum of different COVID-19 severities (Mostafa et al., 2020; Hernández-Terán et al., 2021; Li et al., 2021; Merenstein et al., 2021; Rueca et al., 2021; Ventero et al., 2021). Similar to others, we found that overall URT community composition significantly differed among COVID-19 severity groups (Mostafa et al., 2020; Rueca et al., 2021; Ventero et al., 2021). Interestingly, similar to what has been observed in the URT, gut microbiome community composition was found to be distinct in patients with severe compared with milder presentations of COVID-19 (Yeoh et al., 2021). In the URT, other previous studies have found that patients with mild COVID-19 had an URT microbiome similar to asymptomatic controls, although neither study included patients with severe disease (De Maio et al., 2020; Rosas-Salazar et al., 2021).

While Mostafa et al. observed lower alpha-diversity and richness in patients with COVID-19 compared with those with suspected COVID-19, but who tested negative for the virus, alpha-diversity results were not reported among patients of different severity groups (Mostafa et al., 2020). We found that bacterial richness and alpha-diversity showed a trend toward increasing as disease severity increased; however, alpha-diversity dropped in those patients who had the most severe COVID-19 disease. Rueca et al. also observed that COVID-19 patients in the ICU had a lower richness/alpha-diversity than those with mild or moderate COVID-19 (Rueca et al., 2021). Similarly, the oral microbiome was found to have reduced microbial alpha-diversity in patients with COVID-19, compared with those with non-respiratory diseases, and that alpha-diversity decreased with COVID-19 severity (Soffritti et al., 2021).

We found that *Corynebacterium*_unclassified.ASV0002 relative abundance decreased as COVID-19 severity increased. The species of this ASV could not be definitively identified as it matched with 100% identity to both *C. accolens* and *C. macginleyi*. However, as *C. macginleyi* is usually found in the eye (Sagerfors et al., 2021), while *C. accolens* is a common nose inhabitant (Bomar et al., 2016), we expect this ASV more likely to be *C. accolens.* Similarly, Mostafa et al. found, with whole genome metagenomic sequencing, that *C*. *accolens* incidence was significantly decreased in the URT of patients with SARS-CoV-2 compared with uninfected controls (Mostafa et al., 2020). *Corynebacterium accolens* can inhibit *Streptococcus pneumoniae* and *Staphylococcus aureus* growth, possibly through triolein hydrolysis, which releases oleic acid (Bomar et al., 2016; Menberu et al., 2021). In addition to inhibiting bacterial pathogens, oleic acid is capable of inhibiting other enveloped viruses, such as herpes and influenza (Kohn et al., 1980). Oleoylethanolamide, an oleic acid derivative, may inhibit the release of proinflammatory cytokines induced by SARS-CoV-2 (Ghaffari et al., 2020), potentially reducing the risk of a patient developing a cytokine storm, which is associated with severe disease and high mortality (Hojyo et al., 2020).

In studies examining the URT microbiome in relation to COVID-19 severity, while the specific taxa associated with disease severity have been inconsistent, there has been a trend toward a depletion of commensal bacteria and an increase in known pathogens, in patients with the most severe disease. For example, in our study, we identified a *Corynebacterium* ASV that decreased with COVID-19 severity, while Rueca et al. found that *Bifidobacterium* and *Clostridium* were depleted in those in the ICU (Rueca et al., 2021), and in another study, the most severe COVID-19 patients had reduced *Neisseria*, *Rothia*, and *Prevotella* (Li et al., 2021). In contrast, *Salmonella*, *Scardovia*, *Serratia*, and *Pseudomonadaceae* were more abundant in the nasopharynx of ICU patients compared with those with mild or moderate symptoms, while Ventero et al. found *Alloprevotella*, *Catonella*, *Lachnoanaerobaculum*, *Oribacterium*, multiple *Prevotella*, *Treponema*, and Unclassified *Erysipelotrichaceae* operational taxonomic units (OTUs) to be more abundant and Unclassified *Chloroplast* to be less abundant in patients with severe compared with those with mild COVID-19 (Ventero et al., 2021).

While the lack of consistent bacterial taxa associated with COVID-19 severity is likely partially due to differences in patient location and sequencing/analysis methodologies between studies, potential viral-induced URT microbiome dysbiosis could be contributing to this inconsistency. The “Anna Karenina principle” proposes that stressors, such as viral infection, could have stochastic effects on microbiome community composition (Zaneveld et al., 2017). In our study, we observed that within-group pairwise sample dissimilarities increased as COVID-19 severity increased; those with very severe COVID-19 had URT microbiomes that were the most distinct from each other. An interpretation of these findings could be that we observed increasing microbiome dysbiosis or destabilization as disease severity increased. Other studies have similarly reported respiratory microbiome dysbiosis in patients with COVID-19 compared with controls (Engen et al., 2021; Merenstein et al., 2021) and in those with the most severe disease (Hernández-Terán et al., 2021). SARS-CoV-2-induced instability in the microbiome might make it challenging to identify specific taxa associated with disease severity as the changes could be stochastic and less likely to be shared among different patients, a finding also reported by Li et al., who found that patients with the most severe COVID-19 each had a distinct respiratory microbiome (Li et al., 2021). The SARS-CoV-2-induced URT microbiome dysbiosis itself, rather than consistent changes in specific bacterial taxa, could be associated with an increased risk of severe disease. However, while the overall antibiotic usage in our study was low, in other manuscripts examining the association of respiratory microbiome with COVID-19 severity, patient antibiotic use was either not reported (Mostafa et al., 2020; Rueca et al., 2021; Ventero et al., 2021), reported as high in all groups (Merenstein et al., 2021), or reported as highest in patients with the most severe COVID-19 (Hernández-Terán et al., 2021; Li et al., 2021). Potentially, dysbiosis in some patients could be linked to administration of antibiotics rather than the SARS-CoV-2 infection. However, as we observed a similar URT dysbiosis in spite of having a very low incidence (2/103 patients) of antibiotic use prior to sample collection, this suggests that the virus itself could be associated with dysbiosis.

Our study has numerous strengths, such as a low rate of antibiotic usage allowing us to minimize their confounding effect on the URT microbiome, larger sample size compared with previously published studies, and the inclusion of patients from more than one geographic region and with both severe and mild-to-moderate COVID-19. Furthermore, unlike most prior studies, we included asymptomatic participants uninfected with SARS-CoV-2 as controls. Additionally, all samples were collected during the spring of 2020, thus eliminating seasonal variation in the microbiome and likely co-infection with winter respiratory viruses (e.g., influenza and respiratory syncytial virus). Another strength of our study is that the hospitalized patient swabs were taken at admission, and therefore, we are more likely to have captured the early URT microbiome associated with the development of severe COVID-19 rather than the “hospital microbiome” acquired after a hospital stay. Finally, to our knowledge, we are the first to show an increase in overall bacterial load in severe hospitalized SARS-CoV-2-infected patients compared with mild–moderate outpatients and asymptomatic controls.

However, our study has several limitations. 1) There could be geographical differences in the URT microbiome between patients from New York and Tennessee, and all samples from Tennessee were collected *via* mid-turbinate self-swabs, while the samples from New York were nasopharyngeal samples collected by hospital staff. Similar SARS-CoV-2 viral loads were obtained from mid-turbinate self-swabs and nasopharyngeal swabs collected by healthcare workers from the same patient (Tu et al., 2020); however, the impact of these differing sampling methods on the bacterial load and/or microbiome is unknown. We attempted to control for the influence of geography and sampling method by examining only trends that were consistent across both sites; however, we cannot rule out the roles location or sampling method may play in the URT microbiome. 2) The racial/ethnic makeup differed between groups and race/ethnicity was not recorded for nine participants. We found no strong associations between race/ethnicity and the URT microbiome in infants (Shilts et al., 2015), but to our knowledge, there have not yet been any comprehensive studies examining the association between the URT microbiome and race/ethnicity in adults. 3) The participants from New York were older and had more comorbidities than those from Tennessee; although we adjusted for the effect of race/ethnicity, age, and comorbidities in our ordinal linear regression, it is possible that there was some residual confounding.

Overall, we found trends that changed in the URT microbiome as COVID-19 severity increased, which were consistent across different locations and sampling methods. The URT microbiome could represent a potentially modifiable biomarker/signature for predicting which patients may develop more severe disease. Given our cross-sectional study design, we cannot identify if an altered URT microbiome predisposes patients to more severe COVID-19, or if the URT microbiome changes upon SARS-CoV-2 infection. Further research in additional cohorts is warranted to better understand how the URT microbiome interacts with SARS-CoV-2 to influence disease severity.

## Data Availability Statement

The datasets presented in this study can be found in online repositories. The names of the repository/repositories and accession number(s) can be found in the article/**Supplementary Material**.

## Ethics Statement

The studies involving human participants were reviewed and approved by Vanderbilt University Medical Center IRB. All patients or their surrogate decision makers signed an informed consent at enrollment, which was approved by the IRB at Albert Einstein College of Medicine. The patients/participants provided their written informed consent to participate in this study.

## Author Contributions

MS, CR-S, KK, BS, SR, SM, EP, JT, EJ, and SD contributed to the study design. EJ, MA, VP, MB, DB, MO’N, NA, ES, KK, BW, VG, MF, and JT contributed to the sample collection. MS, BS, HHB, HMB, MA, and SD contributed to the sample processing. CR-S and MS contributed to the statistical analysis. SD and JT obtained the research funding supporting this study. MS, CR-S, and SD wrote the initial version of the manuscript and all authors reviewed and approved the final version.

## Funding

This work was supported by funds from the National Institute of Allergy and Infectious Diseases (under award numbers R21AI142321-02S1, R21AI142321, R21AI154016, and R21AI149262); Centers for Disease Control and Prevention (CDC) 75D3012110094; the National Heart, Lung, and Blood Institute (under award numbers K23HL148638 and R01HL146401); and the Vanderbilt Technologies for Advanced Genomics Core (grant support from the National Institutes of Health under award numbers UL1RR024975, P30CA68485, P30EY08126, and G20RR030956). This research was also supported by NIH/National Center for Advancing Translational Science (NCATS) Einstein-Montefiore CTSA Grant Number UL1 TR002556. The contents are solely the responsibility of the authors and do not necessarily represent the official views of the funding agencies.

## Conflict of Interest

The authors declare that the research was conducted in the absence of any commercial or financial relationships that could be construed as a potential conflict of interest.

## Publisher’s Note

All claims expressed in this article are solely those of the authors and do not necessarily represent those of their affiliated organizations, or those of the publisher, the editors and the reviewers. Any product that may be evaluated in this article, or claim that may be made by its manufacturer, is not guaranteed or endorsed by the publisher.

## Abbreviations

ASV, amplicon sequence variant; BLAST, basic local alignment search tool; CI, confidence interval; COVID-19, coronavirus disease 2019; CT, cycle threshold; ICU, intensive care unit; IRB, Institutional Review Board; NCBI, National Center for Biotechnology Information; NTC, no template control; OR, odds ratio; OTU, operational taxonomic unit; PCoA, principal coordinate analysis; PERMANOVA, permutational analysis of variance; qPCR, quantitative polymerase chain reaction; rRNA, ribosomal ribonucleic acid; RT-qPCR, reverse transcription quantitative real-time PCR; SARS-CoV-2, severe acute respiratory syndrome coronavirus-2; URT, upper respiratory tract; VUMC, Vanderbilt University Medical Center.
