# Supplementary material for: Severe COVID-19 Is Associated With an Altered Upper Respiratory Tract Microbiome
Source: Front Cell Infect Microbiol. 2022 Jan 24;11:781968. doi: 10.3389/fcimb.2021.781968 (PMC8819187; doi:10.3389/fcimb.2021.781968)
Supplement: Supplementary file 1 [file DataSheet_1.docx]

**Title: Severe COVID-19 is associated with an altered upper respiratory tract microbiome.**

Meghan H. Shilts, MHS, MS, ^*^ Christian Rosas-Salazar, MD, MPH,^*^ Britton A. Strickland, Kyle S. Kimura, MD, Mohammad Asad, PhD, Esha Sehanobish, PhD, Michael H. Freeman, MD, Bronson C. Wessinger, Veerain Gupta, Hunter M. Brown, MS, Helen H. Boone, MS, Viraj Patel, MD, Mali Barbi, MD, Danielle Bottalico, MD, MPH, Meaghan O’Neill, PA-C, MPH, Nadeem Akbar, MD, Seesandra V. Rajagopala, MS, PhD, Simon Mallal, MBBS, MD, Elizabeth Phillips, MD, Justin H. Turner, MD, PhD, Elina Jerschow, MD, MS, Suman R. Das, MS, PhD^#^

**Online Repository**

E-Methods Page 2

E-Results Page 5

E-Figure Legends Page 8

E-Tables Page 9

E-References Page 14

**E-Methods**

***Participant clinical and demographic data collection***

Study data were collected and managed using REDCap electronic data capture tools hosted at Vanderbilt University.^1, 2^ REDCap (Research Electronic Data Capture) is a secure, web-based software platform designed to support data capture for research studies, providing 1) an intuitive interface for validated data capture; 2) audit trails for tracking data manipulation and export procedures; 3) automated export procedures for seamless data downloads to common statistical packages; and 4) procedures for data integration and interoperability with external sources.

***Detailed methods***

*DNA extraction*

DNA was extracted with the DNeasy PowerSoil Kit (Qiagen). Mechanical lysis of bacterial cell walls was performed by shaking the samples on a TissueLyser II (Qiagen) for 20 minutes total.

*Assessment of bacterial load*

Equal volumes of extracted DNA were added to duplicate reactions containing universal 16S rRNA primers (UniF340 actcctacgggaggcagcagt, UniR514 attaccgcggctgctggc),^30^ BioRad iQ Supermix, and Invitrogen SYBR DNA stain following the manufacturer’s protocol. Each qPCR plate included a corresponding extraction negative and a no-template negative control (NTC). A serial dilution of standards containing known bacterial copy numbers specific to the primer pair were used as a standard curve as previously described.^30^ PCR reactions were run with a 15 second 95ºC melting and 1 minute 54ºC annealing step for 40 cycles. Cycle threshold (CT) values were plotted against the standard curve to determine copy number. Copy number calculated for each NTC was subtracted from the plate results to adjust for signal generated by primer dimers. Prior to analysis, to approximate a normal distribution and reduce right skewness, bacterial copy number was log transformed [skewness (assessed with the R package *moments*^31^ version 0.14) before transformation=6.10, skewness after transformation=0.10].

*Library construction for 16S rRNA microbiome profiling*

Dual-indexed universal primers appended with Illumina-compatible adapters were used to amplify the hypervariable V4 region of the bacterial 16S rRNA gene.^3^ The PCR mix for each library contained 12.5 µl of MyTaq Mix (Bioline), 0.75 µl DMSO, 1 µl of forward primer, 1 µl of reverse primer, 7 µl of sample, and PCR Certified water (Teknova) was added to achieve a final volume of 25.25 µl. DNA was denatured at 95°C for 2 min, and then 30 cycles of 95°C for 20 seconds, 55°C for 15 seconds, and 72°C were performed. Samples were then incubated at 72°C for 10 min, and samples were held at 4°C until removal from the thermocycler. Each sample was run on a 1% agarose gel to verify reaction success. Libraries were cleaned and normalized with the Invitrogen SequalPrep Kit. After normalization to 1-2 ng/µl, 10 µl of each sample was combined to create the sequencing pool. The pool was cleaned with 1X AMPure XP beads (Beckman Coulter, Brea, California). Libraries were sequenced on an Illumina MiSeq with 2x250 bp reads. A mock community control (ZymoBIOMICS) and 5 extraction and 14 PCR negative controls were run concurrently along with the samples to assess data quality and levels of background contamination.

*Data processing of 16S rRNA microbiome data*

We processed the 16S rRNA sequences using the *dada2* pipeline by following its standard procedure (available at: <https://benjjneb.github.io/dada2/tutorial.html>, last accessed March 9, 2021).^4^ During *filterAndTrim*, the following parameters were used: truncLen=c(240,200), maxN=0, maxEE=c(2,2), truncQ=2, rm.phix=TRUE, compress=TRUE, multithread=TRUE. After reads were merged, as the target fragment size is ~250 bp, sequences >260 or <240 bp were removed, as in our experience, those are likely to be off-target fragments, such as mitochondria. Chimeras were removed with the *removeBimeraDenovo* consensus method. Taxonomy was assigned using the SILVA reference database v138; the *addSpecies* function was used to classify ASVs to species level wherever possible.^5^ Sequences were subsequently processed through the R package *decontam*^6^ to remove any suspected contaminants that were found in the negative control samples. Potential contaminants were detected with the “prevalence” method, in which presence/absence of sequences in the extraction and PCR negative controls is compared to that of real samples. The R package *phyloseq*^7^ version 1.34.0 was used to facilitate data processing.

*Microbiome data analysis*

We conducted unbiased metadata-independent filtering at each taxonomy level by eliminating all taxa that were detected on average <10 times and with an average abundance <0.0005. The absolute counts from the removed taxa were aggregated into a category “other”, which was taken into account when computing simple proportions during data normalization but were otherwise discarded. This was done to reduce the penalty associated with multiple comparisons and to remove likely non-informative data.

Richness and alpha- and beta-diversity metrics were calculated with the R package *vegan*^8^ at the ASV level. All ASVs, regardless of abundance, were included when calculating these diversity indices. To control for differences in sequencing depths, counts were randomly rarefied to the lowest library size of all samples (1,063) and then each microbial ecology index was computed. For each index, this rarefaction and computation process was repeated multiple times (n=400) and the results were averaged. Beta diversity was assessed with the Bray-Curtis dissimilarity index computed on simple proportions and the PermANOVA test as implemented in *Adonis2* ^9^ was used to test for significant differences between overall microbial composition and metadata groupings. As *adonis2* does not accept NA’s (i.e., missing data), only age and sex were added to the model when testing for differences in COVID-19 severity group centroids so that all cases would be included. Principle coordinates analysis (PCoA) was performed in *vegan* in order to ordinate the dissimilarity data and plot it in two dimensional space. PCoA vectors and centroids were extracted with the *betadisper* function. Percent of variance explained by each PCoA axis was calculated by dividing its eigenvalue over the sum of all PCoA eigenvalues and multiplying by 100. Richness and alpha diversity was assessed using Hill numbers N0, N1 and N2, which are, respectively, richness, the exponential of the Shannon index, and the inverted Simpson index.^10^

Microbiome components that were discussed in detail in the manuscript were examined further with a Wilcoxon rank sum test with the Benjamini-Hochberg adjustment for multiple comparisons for all pairwise group comparisons to examine in which specific COVID-19 severity groups the microbiome component was significantly different.

***Ordinal regression modeling***

As we were missing data for the independent variables for some of the samples (N=1 for comorbidities, N=3 for current smoking, N=9 for race/ethnicity, and N=6 for bacterial load), we built two different versions of Model 1 to explore the effects of different ways of handling missing data. In all models, COVID-19 severity was the dependent variable. In Model 1A, the independent variables were age, sex, any comorbidities, current smoking, race/ethnicity, the log of bacterial load, bacterial richness, and the relative abundance of *Corynebacterium*_unclassified.ASV0002 and only those participants with complete data (N=87) were included. For Model 1B, we first imputed the missing values with the *aregImpute* function from *Hmisc* over 20 imputations, using predictive mean matching and the *fit.mult.impute* function was then run to perform ordinal logistic regression.^11^

Due to fundamental data structure differences, the pairwise Bray-Curtis dissimilarities were run separately as Model 2. The COVID-19 severity groups were set as the dependent variable and the within-group dissimilarities as the independent variable. No other independent variables were added to the model as each data point is derived from a comparison between two samples.

For all models, the *validate* and *calibrate* functions from *rms* over 300 bootstrap replications were used to assess model performance.^12^ For Models 1A and 1B, multicollinearity between the independent variables was assessed with *rms*::*vif*.

**E-Results**

***Microbiome quality control assessment***

One ZymoBIOMICS Microbial Community DNA Standard (Zymo), 14 polymerase chain reaction (PCR) negative controls, and 5 extraction negative controls were run concurrently with the URT samples. The mock community control had a community composition very similar to what was expected, with only 9 ASVs being observed >100 times, all of which were exact matches to the expected sequences. The error rate was estimated to be 0.0001. The median (interquartile range) read count of the negative controls was 395 (38-3,558) while for all the URT samples (prior to removal of samples due to low read count) it was 4,674 (1,384-21,986). The negative controls had a significantly lower read count than the URT samples (Wilcoxon rank sum test with continuity correction p-value<0.001). The negative controls also had a significantly different microbial community composition than the URT samples (PERMANOVA *adonis2* *P*<0.001). Sequenced URT samples included in analysis had a median (interquartile) read count of 10,145 (4,411–30,394).

***Pairwise comparisons using a Wilcoxon rank sum test***

Microbiome components that passed our significance threshold after Kruskal-Wallis testing (adjusted P<0.1) and whose medians either consistently increased or decreased as COVID-19 severity increased were examined further. We performed a Wilcoxon rank sum test with the Benjamini-Hochberg adjustment for multiple comparisons for all pairwise group comparisons to examine in which specific COVID-19 severity groups the microbiome component was significantly different. The results for each component are described below.

When we examined bacterial load using quantitative PCR, none of the COVID-19 severity group comparisons were significant (*P* range=0.25–0.94).

For bacterial richness, pairwise comparisons revealed that the uninfected samples were significantly less rich than the moderate, severe, and very severe COVID-19 groups (all adjusted *P*=0.048).

*Corynebacterium*_unclassified.ASV0002 was significantly less abundant in those with mild compared to very severe COVID-19 (adjusted *P*=0.02) and moderate compared to very severe COVID-19 (*P*=0.02), while its abundance in mild compared to severe COVID-19 approached significance (adjusted *P*=0.09).

Within-group pairwise Bray-Curtis dissimilarities were highly significantly different between all severity group comparisons (all adjusted *P*<0.001), except for the mild compared to moderate COVID-19 severity groups, although this comparison was still significantly different (adjusted *P*=0.044).

***Ordinal logistic regression models***

To further examine microbiome parameters that increased or decreased as COVID-19 severity increased, we built three ordinal regression models: 1A) COVID-19 severity groups as the dependent variable and age, sex, race, presence of comorbidities, current smoking, bacterial load, bacterial richness, and relative abundance of *Corynebacterium*_unclassified.ASV0002 as the independent variables with only complete cases, 1B) the same variables as 1A, but imputed values were used, and 3) COVID-19 severity groups as the dependent variable and the pairwise Bray-Curtis dissimilarities between samples within each severity group as the independent variables. The range of variance inflation factors obtained with *rms*::*vif* for Model 1A ranged from 1.07 to 2.07 and for Model 1B from 1.07 to 2.30, indicating low to moderate correlation between the factors, which was not severe enough to warrant corrective measures.

The AUC/concordance index (c-index) for Model 1A was 0.75, 0.772 for Model1B, and 0.66 for Model 2. Model performance was assessed with the *validate* and *calibrate* functions in *rms*. For model 1B, one imputation was used for the *validate* and *calibrate* functions. After running validate, the corrected Somers’ D rank correlation was 0.41 for Model 1A, 0.42 for Model 1B, and 0.32 for Model 2. The full results after validation are available in **Table E4**. After calibration, the mean absolute error rate of Model 1A was 0.08, Model1B was 0.09, and Model 2 was 0.04. The full results of the ordinal logistic regression are available in **Table E5**.

**E-Figure Legends**

**Figure E1**. Flowchart showing participant samples that were included and excluded from the study.

**Figure E2**. Plot of principle coordinates analysis for each of the severity groups along the first axis only (explaining 7.6% of the variance). PCoA vectors were extracted with the *vegan*::*betadisper* function. Each box represents the median and interquartile range, and the mean is shown by the white diamond. Individual points are shown as open circles. Along the first PCoA axis, the severity groups consistently shifted along the same gradient.

**Figure E3**. Plot of effects of variables in Model 1A estimated by ordinal linear regression. Log odds of increased COVID-19 severity are shown on the y-axis for continuous variables (participant age, bacterial load, *Corynebacterium*_unclassified.ASV0002 relative abundance, and bacterial richness) and the x-axis for categorical variables (any comorbidity, current smoker, race/ethnicity, and sex). The chi-square and *P* values are plotted for each variable.

**Figure E4**. Plot of effects of variables in Model 1B estimated by ordinal linear regression. Log odds of increased COVID-19 severity are shown on the y-axis for continuous variables (participant age, bacterial load, Corynebacterium_unclassified.ASV0002 relative abundance, and bacterial richness) and the x-axis for categorical variables (any comorbidity, current smoker, race/ethnicity, and sex). The chi-square and *P* values are plotted for each variable.

**Figure E5**. Plot of effects of variables in Model 2 estimated by ordinal linear regression. Log odds of increased COVID-19 severity are shown on the y-axis, while the Bray-Curtis dissimilarity index between samples is shown on the x-axis. The chi-square and *P* values are plotted on the figure.

**Figure E6**. Within- and between-group pairwise Bray-Curtis dissimilarities are plotted on the y-axis for each of the COVID-19 groups. Larger values indicate that the URT microbial community between the two samples was more dissimilar, while smaller values indicate the opposite. Participants with very severe COVID-19 had URT microbiomes that were very dissimilar to each other, and from participants in the other groups.

**Table E1**. Symptom presence/absence of SARS-CoV-2 infected study participants included in the analysis, stratified by COVID-19 severity. ^*†^

| Symptom | All | COVID mild (n=27) | COVID moderate (n=28) | COVID severe (N=15) | COVID very severe (N=18) | *p*-value^‡^ |
| --- | --- | --- | --- | --- | --- | --- |
| Cough (n=74) | 53 (72%) | 19/25 (76%) | 25/28 (89%) | 6/15 (40%) | 3/6 (50%) | 0.004 |
| Nasal congestion (n=63) | 55 (87%) | 23/27 (85%) | 28/28 (100%) | 3/5 (60%) | 1/3 (33%) | 0.001 |
| Headache (n=64) | 44 (69%) | 13/27 (48%) | 25/28 (89%) | 3/5 (60%) | 3/4 (75%) | 0.011 |
| Sore throat (n=75) | 31 (41%) | 8/27 (30%) | 19/27 (70%) | 2/15 (13%) | 2/6 (33%) | 0.001 |
| Fatigue (n=71) | 56 (79%) | 17/25 (68%) | 25/25 (100%) | 8/15 (53%) | 6/6 (100%) | 0.001 |
| Shortness of breath (n=57) | 28 (49%) | 6/27 (22%) | 21/28 (75%) | 1/1 (100%) | 0/1 (0%) | <0.001 |
| Nausea/vomiting (n=76) | 17 (22%) | 1/27 (4%) | 9/28 (32%) | 5/15 (33% | 2/6 (33%) | 0.038 |
| Diarrhea (n=75) | 24 (32%) | 4/27 (15%) | 13/27 (48%) | 5/15 (33%) | 2/6 (33%) | 0.075 |
| Muscle/joint pain (n=74) | 36 (49%) | 9/26 (35%) | 20/27 (74%) | 4/15 (27%) | 3/6 (50%) | 0.008 |
| Loss of smell or taste (n=75) | 45 (60%) | 12/26 (46%) | 24/28 (86%) | 5/15 (33%) | 4/6 (67%) | 0.002 |
| Problems sleeping (n=60) | 45 (75%) | 15/25 (60%) | 27/28 (96%) | 2/3 (67%) | 1/4 (25%) | 0.002 |

^*^The data are presented as number (%) for categorical variables. As not all symptom data was available for all patients, the denominator is also provided for each age group quartile to indicate for how many patients data on that specific symptom was available.

^†^Data provided in this table is only symptom presence/absence, with no indication of severity, as too few patients from New York indicated symptom severity to make a useful comparison between severity groups. Uninfected control participants were not included as they were not asked to report symptoms.

^‡^*p*-value for the comparison between groups using a Pearson’s chi-squared test.

| **Table E2.** Symptom presence/absence of SARS-CoV-2 infected study participants included in the analysis, stratified by age (years) group quartiles. ^*†^ | | | | | | | | |
| --- | --- | --- | --- | --- | --- | --- | --- | --- |
|  | All (n=83) | 18-30 (n=21) | 31-49 (n=22) | 50-60 (n=20) | 61-93 (n=20) | *p*-value^‡^ |  |  |
| *COVID severity group (n=83)* |  |  |  |  |  | <0.001 |  |  |
| Mild COVID-19 | 27 (33%) | 7 (33%) | 7 (32%) | 5 (25%) | 8 (40%) |  |  |  |
| Moderate COVID-19 | 28 (34% | 12 (57%) | 12 (55%) | 2 (10%) | 2 (10%) |  |  |  |
| Severe COVID-19 | 15 (18%) | 1 (5%) | 2 (9% | 6 (30%) | 6 (30%) |  |  |  |
| Very severe COVID-19 | 13 (16%) | 1 (5%) | 1 (5%) | 7 (35%) | 4 (20%) |  |  |  |
| Symptom | All (n=83) | 18-30 (n=21) | 31-49 (n=22) | 50-60 (n=20) | 61-93 (n=20) | *p*-value^‡^ |  |  |
| Cough (n=74) | 53 (72%) | 17 (81%) | 15/19 (79%) | 10/18 (56%) | 11/16 (69%) | 0.29 |  |  |
| Nasal congestion (n=63) | 55 (87%) | 19/19 (100%) | 15/20 (75%) | 10/12 (83%) | 11/12 (92%) | 0.13 |  |  |
| Headache (n=64) | 44 (69%) | 17/21 (81%) | 12/19 (63%) | 8/11 (73%) | 7/13 (54%) | 0.37 |  |  |
| Sore throat (n=75) | 31 (41%) | 12/20 (60%) | 10/21 (48%) | 4/18 (22%) | 5/16 (31%) | 0.09 |  |  |
| Fatigue (n=71) | 56 (79%) | 14/20 (70%) | 17/19 (89%) | 13/18 (72%) | 12/14 (86%) | 0.38 |  |  |
| Shortness of breath (n=57) | 28 (49%) | 7/19 (37%) | 12/19 (63%) | 3/8 (38%) | 6/11 (55%) | 0.36 |  |  |
| Nausea/vomiting (n=76) | 17 (22%) | 3/21 (14%) | 6/21 (29%) | 5/18 (28%) | 3/16 (19%) | 0.64 |  |  |
| Diarrhea (n=75) | 24 (32%) | 6/20 (30%) | 6/21 (29%) | 9/18 (50%) | 3/16 (19%) | 0.25 |  |  |
| Muscle/joint pain (n=74) | 36 (49%) | 11/21 (52%) | 10/20 (50%) | 8/18 (44%) | 7/15 (47%) | 0.96 |  |  |
| Loss of smell or taste (n=75) | 45 (60%) | 13/20 (65%) | 16/21 (76%) | 10/18 (56%) | 6/16 (38%) | 0.11 |  |  |
| Problems sleeping (n=60) | 45 (75%) | 13/17 (76%) | 17/19 (89%) | 6/11 (55%) | 9/13 (59%) | 0.18 |  |  |
| ^*^The data are presented as number (%) for categorical variables. As not all symptom data was available for all patients, the denominator is also provided for each age group quartile to indicate for how many patients data on that specific symptom was available.  ^†^Data provided in this table is only symptom presence/absence, with no indication of severity, as too few patients from New York indicated symptom severity to make a useful comparison between severity groups. Uninfected control participants were not included as they were not asked to report their symptoms..  ^‡^*p*-value for the comparison between groups using a Pearson’s chi-squared test.  *Definition of abbreviations:* SARS-CoV-2 = Severe acute respiratory syndrome coronavirus-2 | | | | | | | |  |
|  | | | | | | | | |

**Table E3** The full results of Kruskal-Wallis test are presented below. Q-values are Benjamini-Hochberg corrected to adjust for multiple comparisons. The “Magnitude” column is a qualitative assessment of the effect size (eta-squared), provided by the *kruskal_effsize* function in the *rstatix* package.

| ***Microbiome parameter*** | ***p-value*** | ***q-value*** | ***n*** | ***Eta-squared (CI)*** | ***Magnitude*** |
| --- | --- | --- | --- | --- | --- |
| Dissimilarity | <0.001 | <0.001 | 1102 | 0.16 (0.13-0.21) | large |
| Richness | 0.01 | 0.05 | 103 | 0.09 (0.01-0.27) | moderate |
| Shannon | 0.18 | 0.32 | 103 | 0.02 (-0.02-0.19) | small |
| Simpson | 0.36 | 0.52 | 103 | <0.001 (-0.03-0.17) | small |
| Bacterial load | 0.1 | 0.23 | 103 | 0.04 (-0.02-0.24) | small |
| *Staphylococcus unclassified ASV0001* | 0.17 | 0.31 | 103 | 0.02 (-0.02-0.21) | small |
| *Corynebacterium unclassified ASV0002* | 0.009 | 0.048 | 103 | 0.10 (0.03-0.26) | moderate |
| *Corynebacterium unclassified ASV0003* | 0.12 | 0.24 | 103 | 0.03 (-0.01-0.18) | small |
| *Dolosigranulum pigrum ASV0006* | 0.04 | 0.12 | 103 | 0.06 (0.02-0.2) | moderate |
| *Corynebacterium unclassified ASV0004* | 0.009 | 0.05 | 103 | 0.1 (0.03-0.26) | moderate |
| *Lawsonella clevelandensis ASV0010* | <0.001 | 0.001 | 103 | 0.2 (0.09-0.38) | large |
| *Peptoniphilus unclassified unclassified ASV0007* | 0.02 | 0.07 | 103 | 0.08 (0.02-0.24) | moderate |
| *Streptococcus unclassified ASV0008* | 0.11 | 0.24 | 103 | 0.04 (-0.01-0.21) | small |
| *Corynebacterium unclassified ASV0011* | 0.77 | 0.8 | 103 | -0.02 (-0.03-0.08) | small |
| *Moraxella unclassified ASV0017* | 0.67 | 0.75 | 103 | -0.02 (-0.03-0.12) | small |
| *Streptococcus unclassified ASV0009* | 0.55 | 0.66 | 103 | -0.01 (-0.03-0.14) | small |
| *Corynebacterium unclassified ASV0078* | 0.01 | 0.06 | 103 | 0.09 (-0.0096-0.28) | moderate |
| *Neisseriaceae unclassified unclassified ASV0046* | 0.19 | 0.32 | 103 | 0.02 (-0.01-0.15) | small |
| *Moraxella unclassified ASV0013* | 0.4 | 0.53 | 103 | <0.001 (-0.02-0.12) | small |
| *Staphylococcus unclassified ASV0056* | 0.09 | 0.22 | 103 | 0.04 (-0.007-0.24) | small |
| *Klebsiella unclassified ASV0005* | 0.61 | 0.69 | 103 | -0.01 (-0.03-0.12) | small |
| *Bacteroides vulgatus ASV0096* | 0.29 | 0.43 | 103 | 0.01 (-0.02-0.15) | small |
| *Finegoldia unclassified unclassified ASV0012* | 0.42 | 0.54 | 103 | <0.001 (-0.03-0.14) | small |
| *Veillonella unclassified ASV0016* | 0.07 | 0.17 | 103 | 0.05 (-0.02-0.29) | small |
| *Blautia unclassified ASV0067* | 0.15 | 0.28 | 103 | 0.03 (-0.02-0.22) | small |
| *Cutibacterium unclassified ASV0028* | <0.001 | <0.001 | 103 | 0.22 (0.11-0.39) | large |
| *Anaerococcus unclassified unclassified ASV0018* | 0.06 | 0.16 | 103 | 0.05 (-0.01-0.21) | small |
| *Lactobacillus iners ASV0127* | 0.05 | 0.14 | 103 | 0.06 (-0.00053-0.19) | small |
| *Pseudomonas unclassified ASV0039* | 0.69 | 0.76 | 103 | -0.02 (-0.02-0.15) | small |
| *Anaerococcus unclassified unclassified ASV0022* | 0.12 | 0.25 | 103 | 0.03 (-0.01-0.19) | small |
| *Corynebacterium unclassified ASV0015* | 0.002 | 0.02 | 103 | 0.13 (-0.007-0.41) | moderate |
| *Enterococcus cecorum ASV0099* | <0.001 | <0.001 | 103 | 0.54 (0.38-0.74) | large |
| *Prevotella melaninogenica ASV0027* | 0.12 | 0.24 | 103 | 0.03 (-0.01-0.21) | small |
| *Planococcaceae unclassified unclassified ASV0139* | <0.001 | <0.001 | 103 | 0.47 (0.29-0.72) | large |
| *Neisseriaceae unclassified unclassified ASV0060* | 0.4 | 0.53 | 103 | <0.001 (-0.03-0.13) | small |
| *Haemophilus unclassified ASV0020* | 0.46 | 0.59 | 103 | 0.004 (-0.03-0.14) | small |
| *Acinetobacter unclassified ASV0065* | 0.75 | 0.8 | 103 | -0.02 (-0.03-0.08) | small |
| *Burkholderia-Caballeronia-Paraburkholderia unclassified ASV0083* | <0.001 | <0.001 | 103 | 0.33 (0.15-0.59) | large |
| *Staphylococcus haemolyticus ASV0040* | 0.17 | 0.31 | 103 | 0.02 (-0.02-0.23) | small |
| *Streptococcus unclassified ASV0031* | 0.003 | 0.03 | 103 | 0.12 (0.03-0.33) | moderate |
| *Corynebacterium unclassified ASV0076* | 0.33 | 0.48 | 103 | 0.01 (-0.02-0.12) | small |
| *Corynebacterium unclassified ASV0052* | 0.99 | 0.99 | 103 | -0.04 (-0.04-0.07) | small |
| *Corynebacterium jeikeium ASV0045* | 0.007 | 0.05 | 103 | 0.1 (0.01-0.33) | moderate |
| *Corynebacterium unclassified ASV0059* | 0.78 | 0.8 | 103 | -0.02 (-0.03-0.05) | small |
| *Mycoplasma salivarium ASV0170* | <0.001 | 0.004 | 103 | 0.17 (0.04-0.45) | large |
| *Neisseriaceae unclassified unclassified ASV0073* | 0.04 | 0.13 | 103 | 0.06 (-0.0075-0.24) | small |
| *Chloroplast unclassified unclassified ASV0075* | 0.49 | 0.61 | 103 | -0.01 (-0.02-0.1) | small |
| *Actinomyces odontolyticus ASV0037* | 0.009 | 0.05 | 103 | 0.1 (0.02-0.32) | moderate |
| *Enhydrobacter unclassified ASV0093* | 0.13 | 0.26 | 103 | 0.03 (-0.01-0.19) | small |
| *Corynebacterium kroppenstedtii ASV0054* | 0.21 | 0.33 | 103 | 0.02 (-0.0051-0.14) | small |
| *Veillonella unclassified ASV0029* | 0.04 | 0.12 | 103 | 0.06 (-0.0073-0.26) | moderate |
| *Lactococcus unclassified ASV0094* | 0.01 | 0.05 | 103 | 0.1 (0.03-0.24) | moderate |
| *Prevotella melaninogenica ASV0030* | 0.01 | 0.06 | 103 | 0.09 (0.01-0.31) | moderate |
| *Prevotella salivae ASV0051* | 0.007 | 0.05 | 103 | 0.1 (0.02-0.3) | moderate |
| *Chloroplast unclassified unclassified ASV0109* | 0.58 | 0.68 | 103 | -0.01 (-0.02-0.11) | small |
| *Anaerococcus unclassified unclassified ASV0061* | 0.24 | 0.37 | 103 | 0.02 (-0.01-0.14) | small |
| *Anaerococcus unclassified unclassified ASV0047* | 0.86 | 0.88 | 103 | -0.03 (-0.03-0.08) | small |
| *Planococcaceae unclassified unclassified ASV0168* | 0.03 | 0.09 | 103 | 0.07 (0.0097-0.28) | moderate |
| *Burkholderia.Caballeronia.Paraburkholderia unclassified ASV0146* | 0.003 | 0.03 | 103 | 0.12 (0.01-0.4) | moderate |
| *Campylobacter ureolyticus ASV0042* | 0.49 | 0.61 | 103 | -0.01 (-0.03-0.13) | small |
| *Prevotella buccalis ASV0091* | 0.78 | 0.8 | 103 | -0.02 (-0.03-0.09) | small |
| *Lactobacillus unclassified ASV0175* | 0.15 | 0.28 | 103 | 0.03 (-0.0051-0.22) | small |
| *Prevotella unclassified ASV0231* | 0.007 | 0.05 | 103 | 0.1 (0.01-0.34) | moderate |
| *Prevotella timonensis ASV0068* | 0.61 | 0.69 | 103 | -0.01 (-0.02-0.1) | small |
| *Megasphaera micronuciformis ASV0055* | 0.002 | 0.02 | 103 | 0.13 (0.03-0.36) | moderate |
| *Granulicatella unclassified ASV0070* | 0.02 | 0.09 | 103 | 0.07 (0.02-0.25) | moderate |
| *Gemella unclassified ASV0044* | 0.73 | 0.79 | 103 | -0.02 (-0.03-0.11) | small |
| *Anaerostipes hadrus ASV0172* | 0.02 | 0.07 | 103 | 0.08 (0.0013-0.28) | moderate |
| *Neisseria unclassified ASV0043* | 0.36 | 0.52 | 103 | 0.003 (-0.02-0.18) | small |
| *Staphylococcus pettenkoferi ASV0155* | 0.21 | 0.33 | 103 | 0.02 (-0.00097-0.18) | small |
| *Prevotella histicola ASV0063* | 0.03 | 0.09 | 103 | 0.07 (-0.00064-0.29) | moderate |
| *Anaerococcus unclassified unclassified ASV0036* | 0.14 | 0.27 | 103 | 0.03 (0.0043-0.25) | small |
| *Staphylococcus unclassified ASV0118* | 0.38 | 0.52 | 103 | 0.002 (-0.01-0.13) | small |
| *Veillonella unclassified ASV0032* | 0.06 | 0.16 | 103 | 0.05 (-0.0034-0.24) | small |
| *Staphylococcus pettenkoferi ASV0158* | 0.21 | 0.33 | 103 | 0.02 (-0.0028-0.19) | small |
| *Prevotella unclassified ASV0281* | 0.02 | 0.09 | 103 | 0.07 (0.01-0.26) | moderate |
| *Corynebacterium unclassified ASV0080* | 0.15 | 0.28 | 103 | 0.03 (-0.01-0.15) | small |
| *Proteus unclassified ASV0041* | 0.05 | 0.14 | 103 | 0.06 (-0.0023-0.29) | small |
| *Alloprevotella tannerae ASV0113* | 0.72 | 0.79 | 103 | -0.02 (-0.02-0.13) | small |
| *Micrococcaceae unclassified unclassified ASV0206* | 0.11 | 0.24 | 103 | 0.04 (-0.02-0.23) | small |
| *Capnocytophaga leadbetteri ASV0053* | 0.006 | 0.05 | 103 | 0.11 (0.02-0.32) | moderate |
| *Veillonella unclassified ASV0064* | 0.09 | 0.22 | 103 | 0.04 (-0.0025-0.23) | small |
| *Enterobacteriaceae unclassified unclassified ASV0048* | 0.9 | 0.9 | 103 | -0.03 (-0.03-0.08) | small |
| *Fusobacterium periodonticum ASV0057* | 0.53 | 0.64 | 103 | -0.01 (-0.02-0.15) | small |
| *Prevotella unclassified ASV0208* | 0.37 | 0.52 | 103 | 0.003 (-0.02-0.13) | small |
| *Lactobacillus unclassified ASV0035* | 0.21 | 0.33 | 103 | 0.02 (-0.0084-0.14) | small |
| *Rothia mucilaginosa ASV0072* | 0.05 | 0.14 | 103 | 0.06 (-0.01-0.26) | small |
| *Corynebacterium unclassified ASV0077* | 0.3 | 0.45 | 103 | 0.01 (-0.02-0.17) | small |
| *Streptococcus unclassified ASV0074* | 0.41 | 0.54 | 103 | <0.001 (-0.02-0.18) | small |
| *Rothia dentocariosa ASV0025* | 0.07 | 0.17 | 103 | 0.05 (-0.01-0.25) | small |
| *Fenollaria unclassified unclassified ASV0149* | 0.52 | 0.63 | 103 | -0.01 (-0.02-0.09) | small |
| *Streptococcus unclassified ASV0079* | 0.02 | 0.07 | 103 | 0.08 (-0.0035-0.31) | moderate |
| *Actinomyces graevenitzii ASV0085* | 0.21 | 0.33 | 103 | 0.02 (-0.01-0.21) | small |
| *Streptococcus unclassified ASV0082* | 0.19 | 0.32 | 103 | 0.02 (-0.01-0.14) | small |
| *Staphylococcus unclassified ASV0186* | 0.59 | 0.69 | 103 | -0.01 (-0.02-0.06) | small |
| *Prevotella nanceiensis ASV0151* | 0.39 | 0.53 | 103 | 0.002 (-0.02-0.16) | small |
| *Peptostreptococcus stomatis ASV0173* | 0.05 | 0.14 | 103 | 0.05 (-0.01-0.29) | small |
| *Dolosigranulum unclassified ASV0242* | 0.61 | 0.69 | 103 | -0.01 (-0.02-0.07) | small |
| *Actinomyces unclassified ASV0069* | 0.48 | 0.61 | 103 | -0.01 (-0.03-0.14) | small |
| *Pasteurellaceae unclassified unclassified ASV0182* | 0.001 | 0.02 | 103 | 0.14 (0.01-0.37) | large |

**Table E4**. Validation results over 300 bootstraps of ordinal logistic regression Models 1A, 1B, and 2.

|  | **Model 1A** | | | | | **Model 1B** | | | | | **Model 2** | | | | |
| --- | --- | --- | --- | --- | --- | --- | --- | --- | --- | --- | --- | --- | --- | --- | --- |
| *Index name^*^* | *Index original* | *training* | *test* | *optimism* | *Index corrected* | *Index original* | *training* | *test* | *optimism* | *Index corrected* | *Index original* | *training* | *test* | *optimism* | *Index corrected* |
| *Dxy* | 0.53 | 0.59 | 0.46 | 0.12 | 0.41 | 0.52 | 0.58 | 0.47 | 0.1 | 0.42 | 0.32 | 0.32 | 0.32 | 0 | 0.32 |
| *R^2^* | 0.43 | 0.52 | 0.33 | 0.18 | 0.25 | 0.42 | 0.5 | 0.35 | 0.15 | 0.27 | 0.14 | 0.14 | 0.14 | 0 | 0.13 |
| *Intercept* | 0 | 0 | 0.04 | -0.04 | 0.04 | 0 | 0 | 0.01 | -0.01 | 0.01 | 0 | 0 | 0 | 0 | 0 |
| *Slope* | 1 | 1 | 0.66 | 0.34 | 0.66 | 1 | 1 | 0.7 | 0.3 | 0.7 | 1 | 1 | 1 | 0 | 1 |
| *Emax* | 0 | 0 | 0.1 | 0.1 | 0.1 | 0 | 0 | 0.08 | 0.08 | 0.08 | 0 | 0 | 0 | 0 | 0 |
| *D* | 0.52 | 0.68 | 0.38 | 0.3 | 0.22 | 0.5 | 0.64 | 0.39 | 0.25 | 0.26 | 0.14 | 0.14 | 0.14 | 0 | 0.13 |
| *U* | -0.02 | -0.02 | -1.36 | 1.33 | -1.36 | -0.02 | -0.02 | -1.43 | 1.41 | -1.43 | 0 | 0 | -1.43 | 1.43 | -1.43 |
| *Q* | 0.54 | 0.7 | 1.74 | -1.04 | 1.58 | 0.52 | 0.66 | 1.82 | -1.16 | 1.68 | 0.14 | 0.14 | 1.57 | -1.43 | 1.57 |
| *B* | 0.2 | 0.19 | 0.23 | -0.04 | 0.24 | 0.2 | 0.19 | 0.22 | -0.03 | 0.23 | 0.23 | 0.23 | 0.23 | 0 | 0.24 |

^*^Explanation of indices: Dxy is Somers’ D rank correlation; R^2^, intercept and slope are derived from the overall logistic calibration equation; Emax is the maximum absolute difference in predicted and calibrated probabilities; D is the discrimination index (model L.R. (chi-square – 1)/n); U is the unreliability index, which is the difference in -2 log likelihood between un-calibrated X beta and X beta with overall intercept and slope calibrated to test sample/n; Q is overall quality index (logarithmic probability score),;B is Brier or quadratic probability score.

**Table E5.** Ordinal logistic regression results of Models 1A and 1B.

|  |  | **Model 1A** | | | | | **Model 1B** | | | |
| --- | --- | --- | --- | --- | --- | --- | --- | --- | --- | --- |
|  |  | *Odds ratio (95% CI)* | *Chi-Square* | *d.f.* | *P* | *Odds ratio (95% CI)* | | *Chi-Square* | *d.f.* | *P* |
| Age |  | 1.2 (0.55-2.59) | 0.21 | 1 | 0.65 | 1.59 (0.8-3.14) | | 1.76 | 1 | 0.18 |
| Sex | Female:Male | 1.51 (0.67-3.38) | 0.98 | 1 | 0.32 | 1.65 (0.77-3.52) | | 1.65 | 1 | 0.2 |
| Any comorbidity | Yes:No | 1.9 (0.77-4.69) | 1.95 | 1 | 0.16 | 1.96 (0.84-4.56) | | 2.46 | 1 | 0.12 |
| Current smoker | Yes:No | 0.61 (0.07-5.07) | 0.21 | 1 | 0.65 | 0.87 (0.16-4.65) | | 0.03 | 1 | 0.87 |
| Race | Black:White | 5.31 (1.24-22.73) | 10.3 | 3 | 0.02 | 4.29 (1.11-16.61) | | 12.38 | 3 | 0.01 |
|  | Hispanic:White | 10.8 (2.29-50.83) | -- | -- | -- | 10.78 (2.77-41.99) | | -- | -- | -- |
|  | Other:White | 3.19 (0.48-21.04) | -- | -- | -- | 1.81 (0.3-10.94) | | -- | -- | -- |
| Bacterial load |  | 2.09 (1.04-4.21) | 4.23 | 1 | 0.04 | 1.71 (0.88-3.31) | | 2.5 | 1 | 0.11 |
| Richness |  | 1.51 (0.96-2.37) | 3.17 | 1 | 0.07 | 1.61 (1.04-2.51) | | 4.53 | 1 | 0.03 |
| *Corynebacterium* unclassified ASV0002 |  | 0.69 (0.43-1.09) | 2.55 | 1 | 0.11 | 0.66 (0.43-1.01) | | 3.61 | 1 | 0.06 |
| **TOTAL** | **--** | **--** | **36.69** | **10** | **<0.001** | **--** | | **42.62** | **10** | **<0.001** |

**References**

1. Harris PA, Taylor R, Minor BL, Elliott V, Fernandez M, O'Neal L, et al. The REDCap consortium: Building an international community of software platform partners. J Biomed Inform 2019; 95:103208.

2. Harris PA, Taylor R, Thielke R, Payne J, Gonzalez N, Conde JG. Research electronic data capture (REDCap)--a metadata-driven methodology and workflow process for providing translational research informatics support. J Biomed Inform 2009; 42:377-81.

3. Kozich JJ, Westcott SL, Baxter NT, Highlander SK, Schloss PD. Development of a dual-index sequencing strategy and curation pipeline for analyzing amplicon sequence data on the MiSeq Illumina sequencing platform. Appl Environ Microbiol 2013; 79:5112-20.

4. Callahan BJ, McMurdie PJ, Rosen MJ, Han AW, Johnson AJ, Holmes SP. DADA2: High-resolution sample inference from Illumina amplicon data. Nat Methods 2016; 13:581-3.

5. Pruesse E, Quast C, Knittel K, Fuchs BM, Ludwig W, Peplies J, et al. SILVA: a comprehensive online resource for quality checked and aligned ribosomal RNA sequence data compatible with ARB. Nucleic Acids Res 2007; 35:7188-96.

6. Davis NM, Proctor DM, Holmes SP, Relman DA, Callahan BJ. Simple statistical identification and removal of contaminant sequences in marker-gene and metagenomics data. Microbiome 2018; 6:226.

7. McMurdie PJ, Holmes S. phyloseq: an R package for reproducible interactive analysis and graphics of microbiome census data. PLoS One 2013; 8:e61217.

8. Oksanen J, Blanchet FG, Kindt R, Legendre P, Minchin PR, O'Hara RB, et al. vegan: Community Ecology Package. R package version 2.0-10. 2014.

9. Anderson MJ. A new method for non-parametric multivariate analysis of variance. Austral Ecol 2001; 26:32-46.

10. Hill MO. Diversity and evenness: a unifying notation and its consequences. Ecology 1973; 54:427-32.

11. Harrell FE. , with contributions from Charles Dupont and many others. Hmisc: Harrell Miscellaneous. R package version 4.4-2. <https://CRAN.R-project.org/package=Hmisc>. 2020.

12. rms: Regression Modeling Strategies. R package version 6.2-0. 2021.] Available from <https://CRAN.R-project.org/package=rms>.
