# Supplementary figures and images for: Severe COVID-19 Is Associated With an Altered Upper Respiratory Tract Microbiome
Source: Front Cell Infect Microbiol. 2022 Jan 24;11:781968. doi: 10.3389/fcimb.2021.781968 (PMC8819187; doi:10.3389/fcimb.2021.781968)

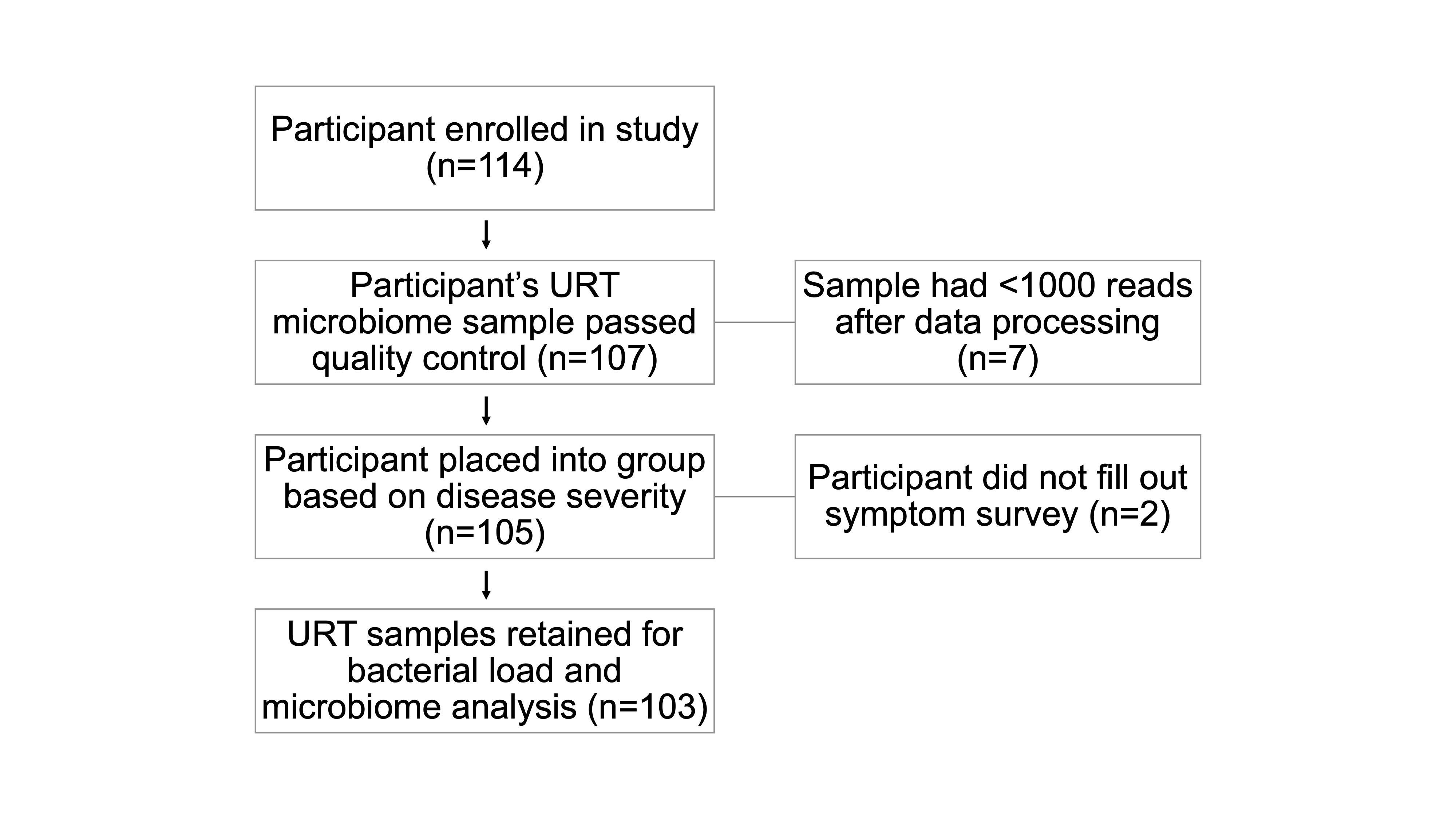

Supplement: Supplementary file 2 [file Image_1.jpeg]

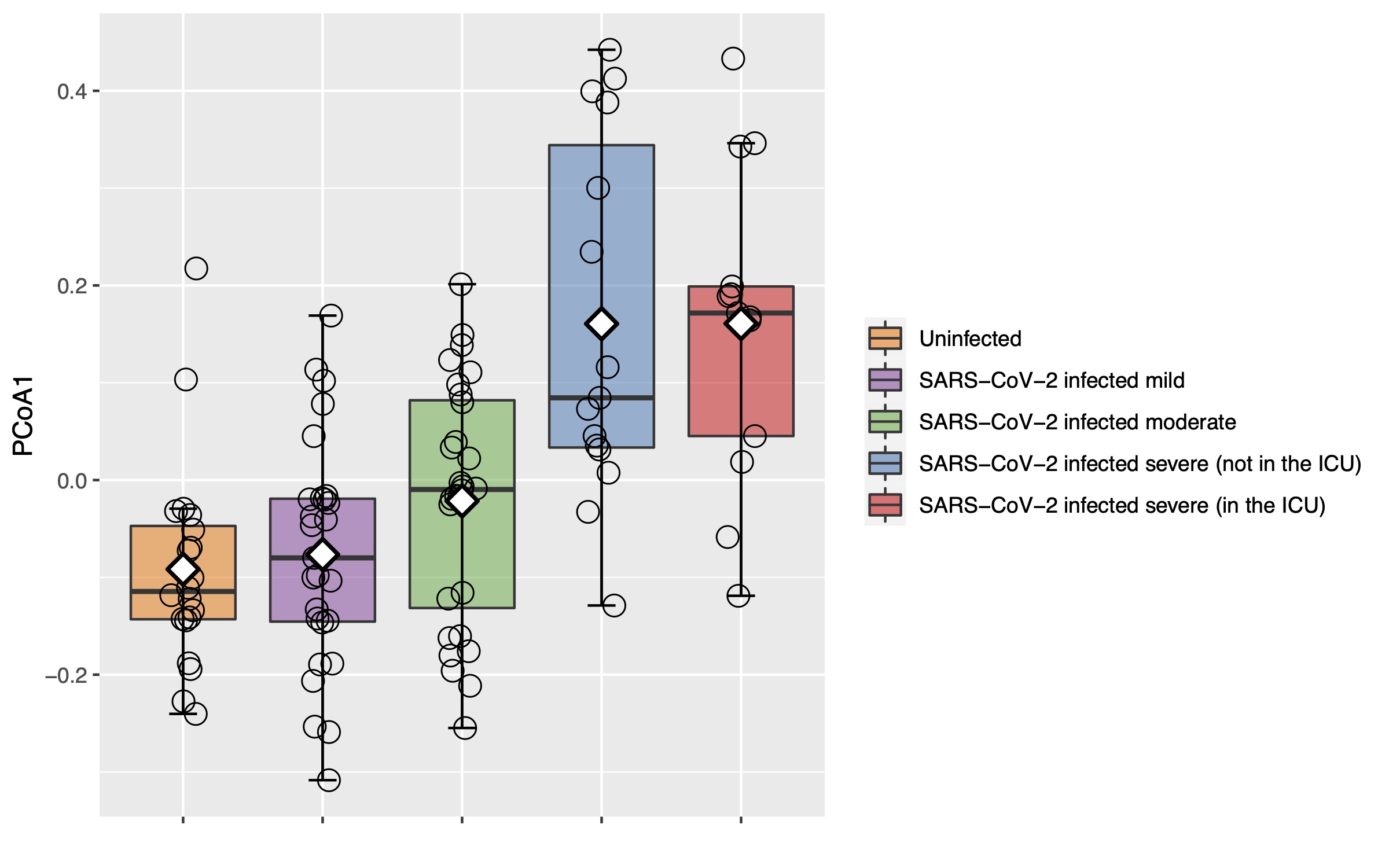

Supplement: Supplementary file 3 [file Image_2.jpeg]

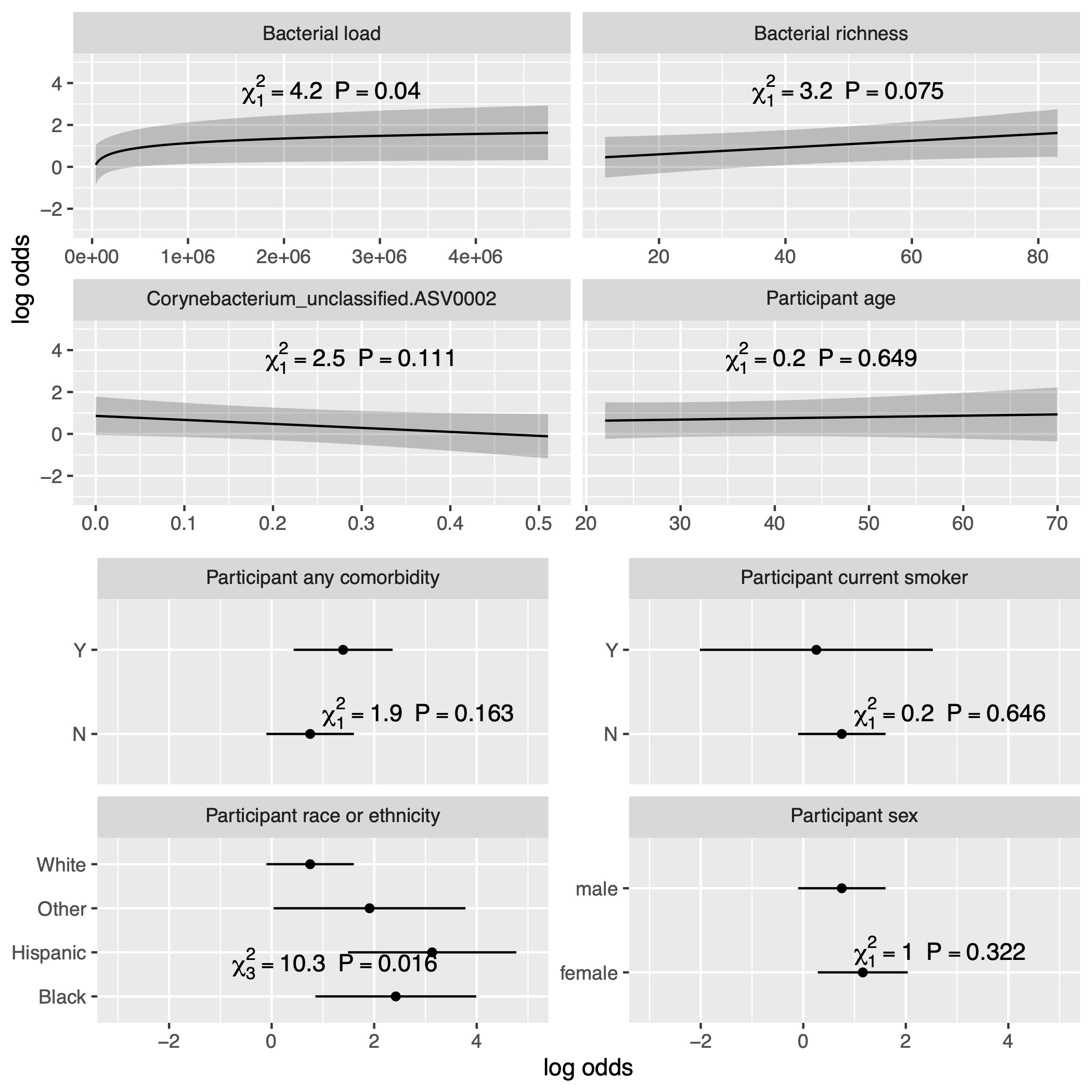

Supplement: Supplementary file 4 [file Image_3.jpeg]

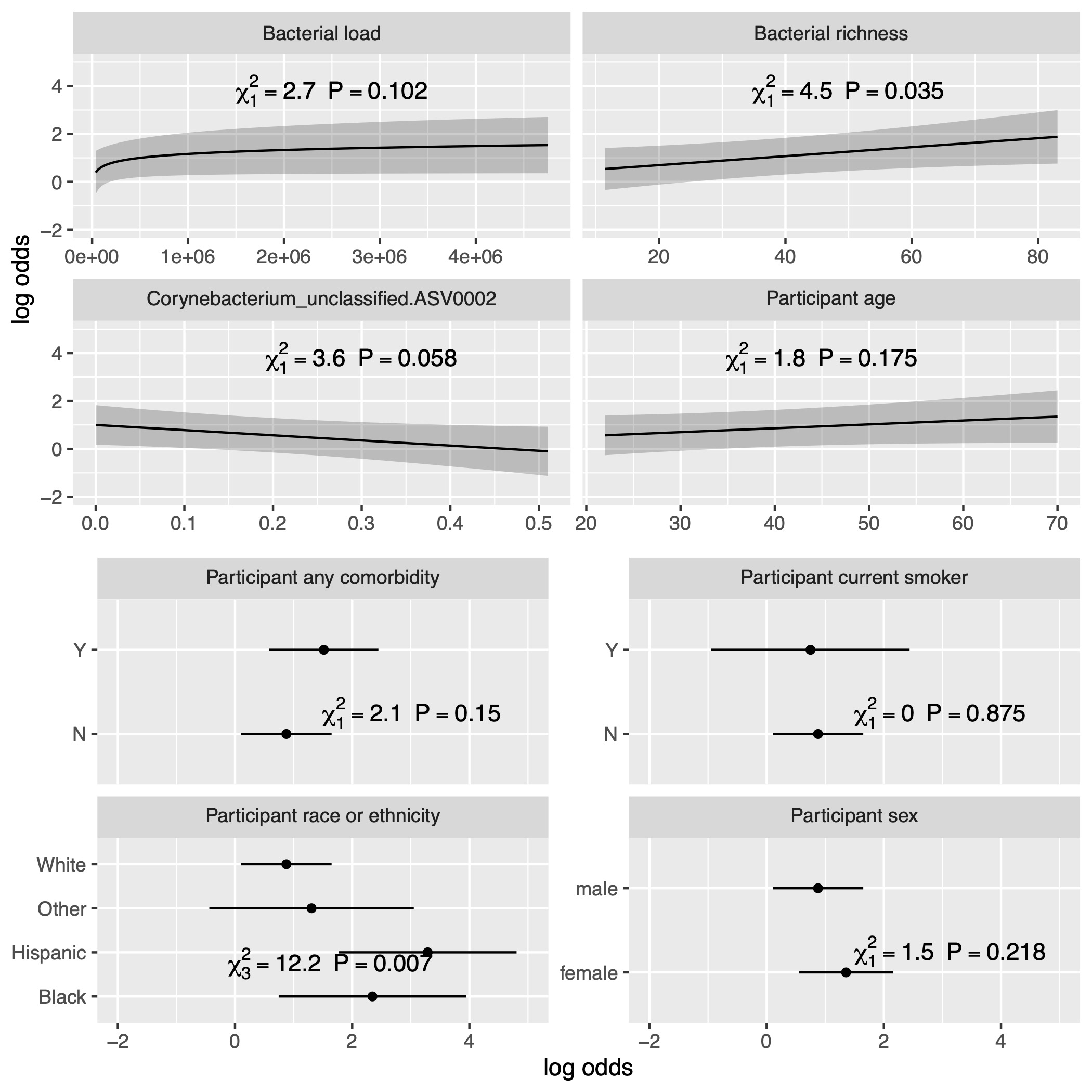

Supplement: Supplementary file 5 [file Image_4.jpeg]

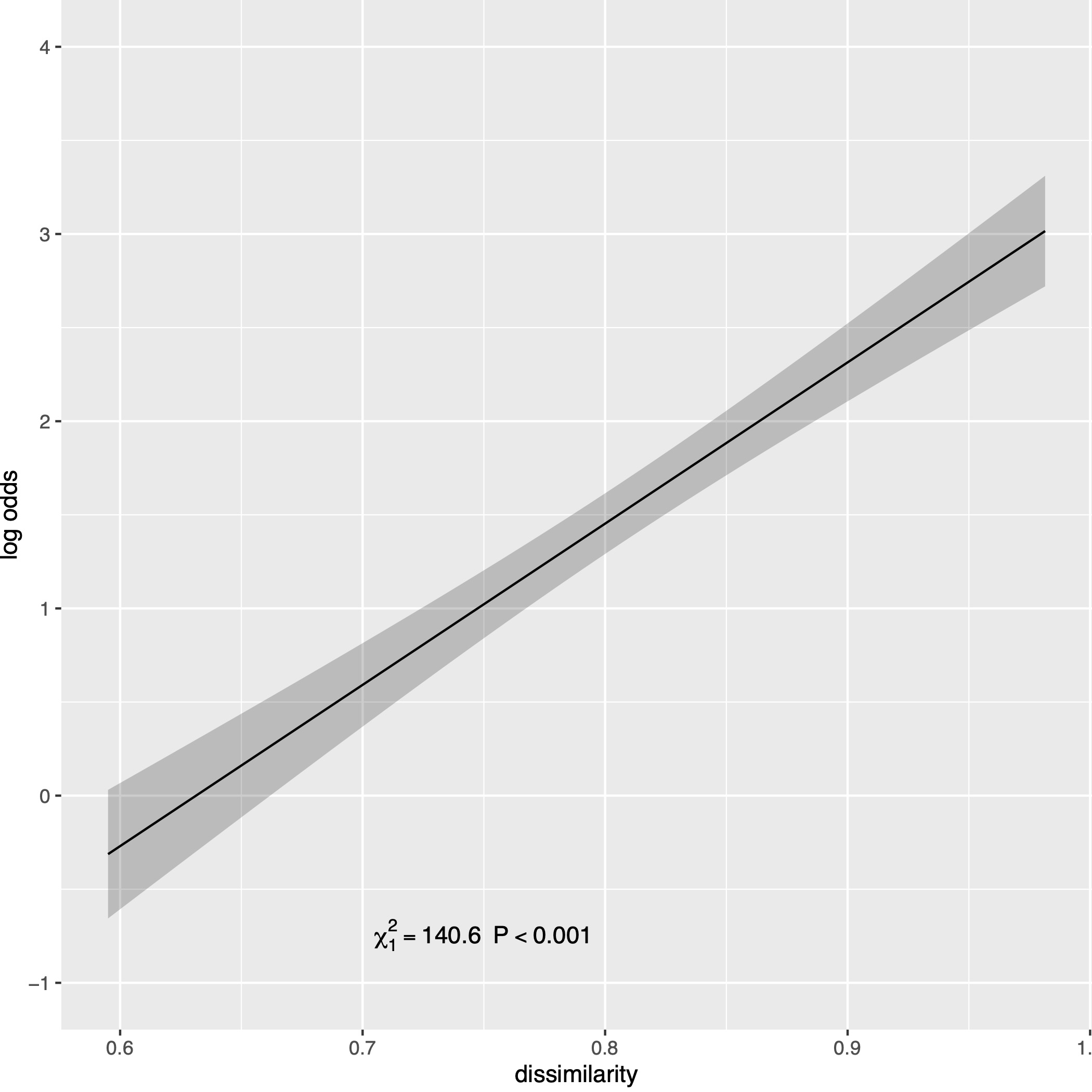

Supplement: Supplementary file 6 [file Image_5.jpeg]

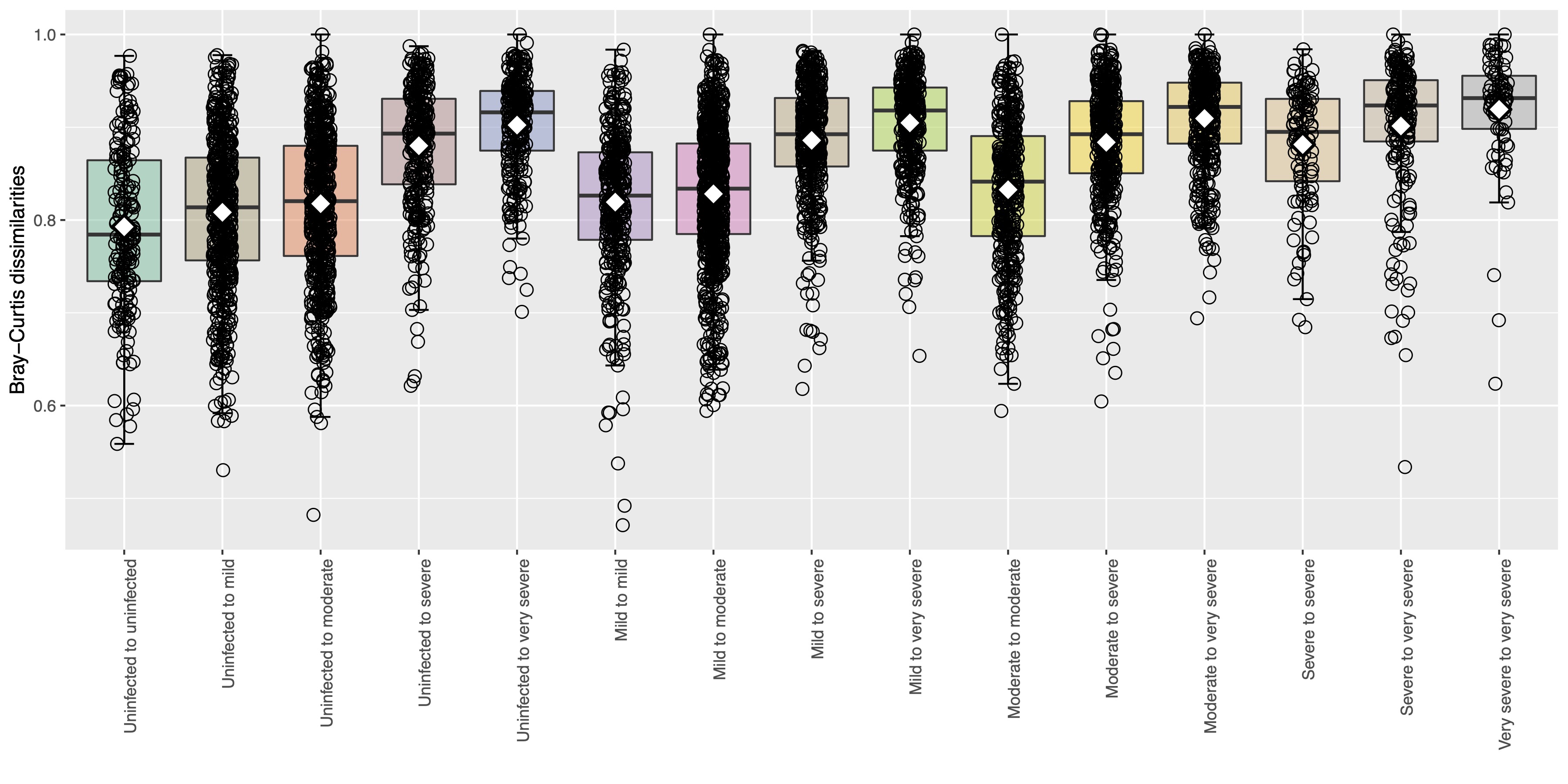

Supplement: Supplementary file 7 [file Image_6.jpeg]
